# Supplementary figures and images for: Quantitative analysis of massive SARS-CoV-2 testing in the community in France in 2021–2022 reveals the associations of variant, vaccination, and age with viral dynamics in symptomatic individuals
Source: PLoS Comput Biol. 2026 Jul 27;22(7):e1013811. doi: 10.1371/journal.pcbi.1013811 (PMC13426954; doi:10.1371/journal.pcbi.1013811)

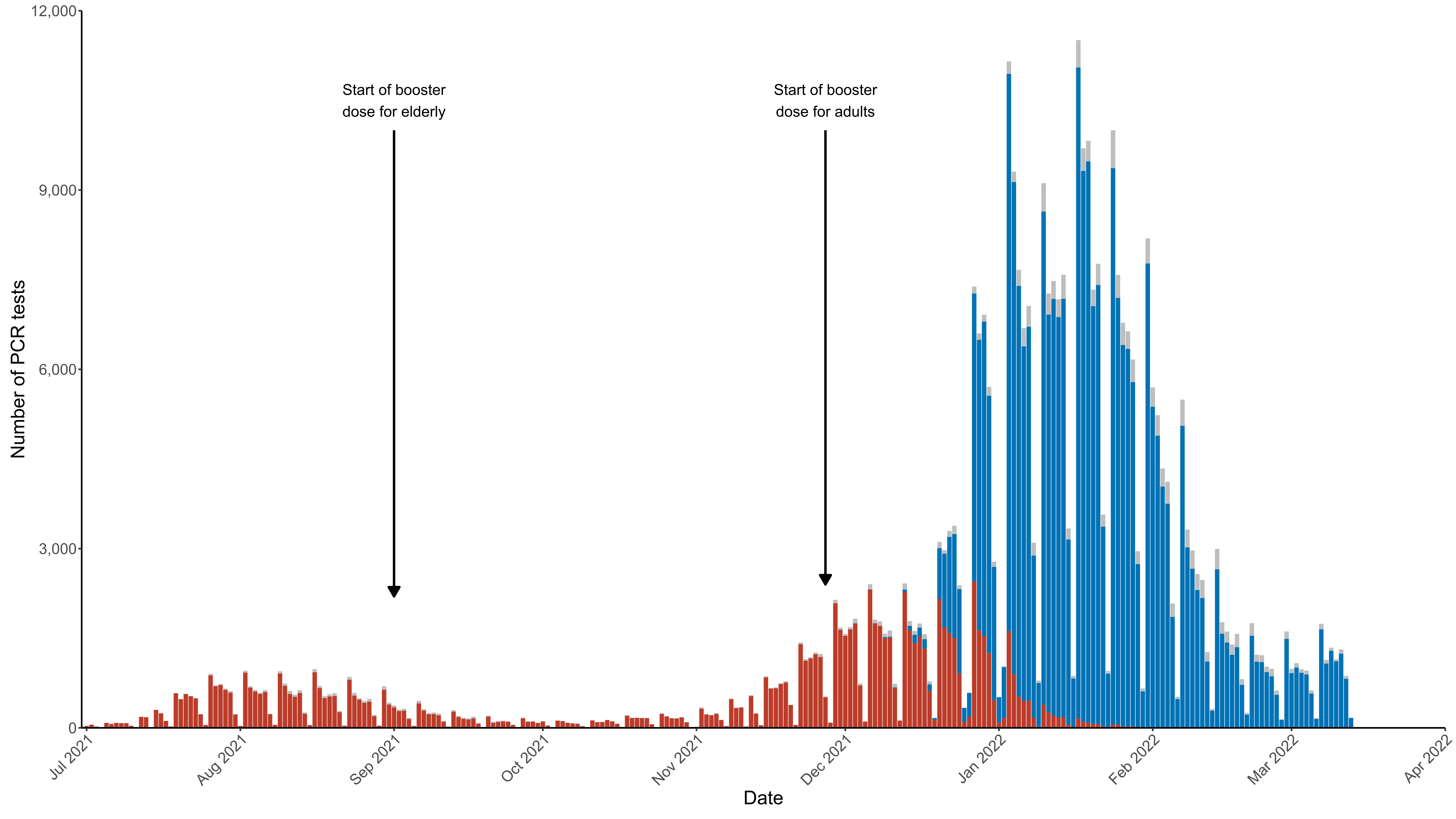

Supplement: S1 Fig — (TIFF) [file pcbi.1013811.s001.tiff]

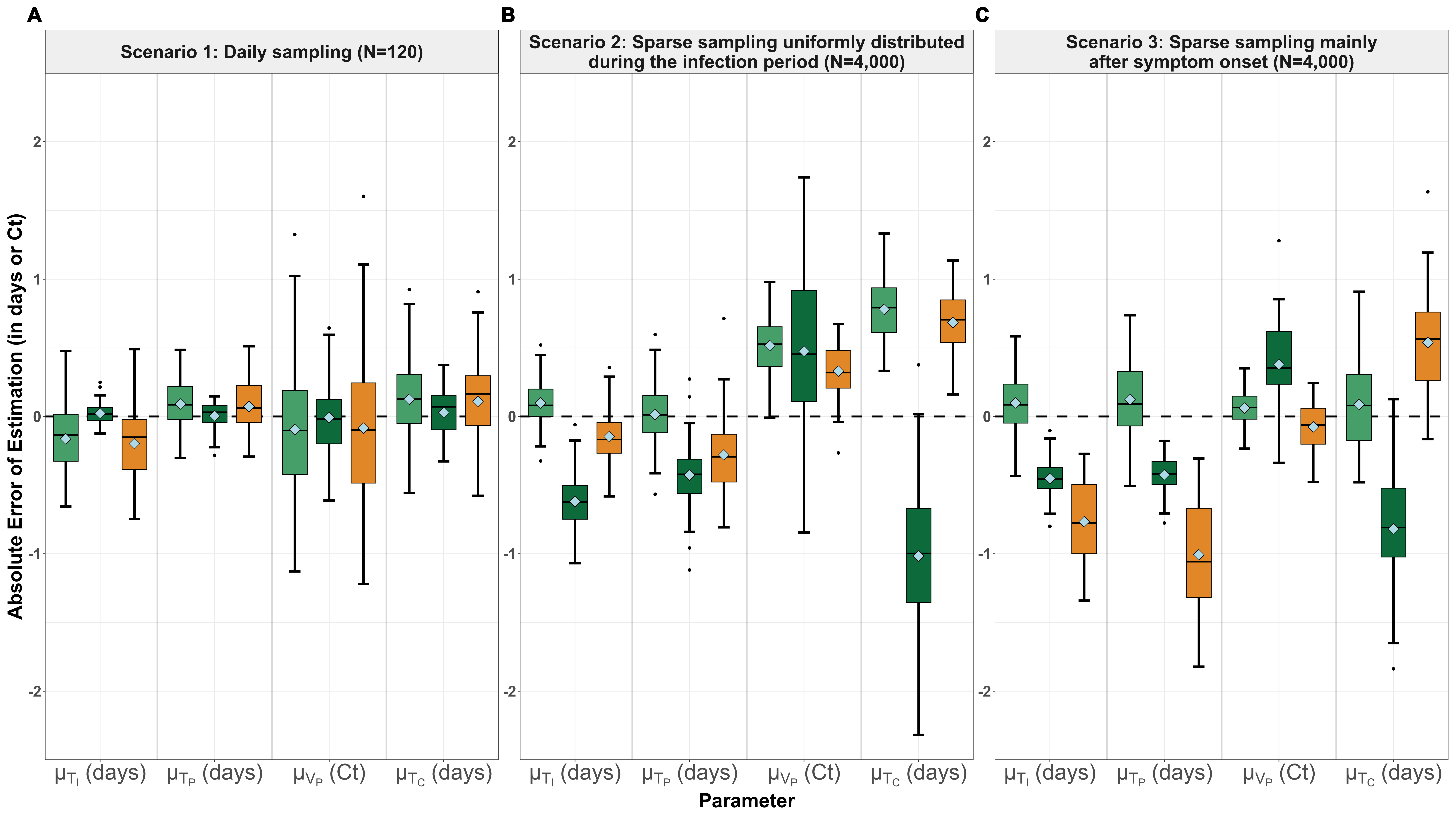

Supplement: S5 Fig — (TIFF) [file pcbi.1013811.s007.tiff]

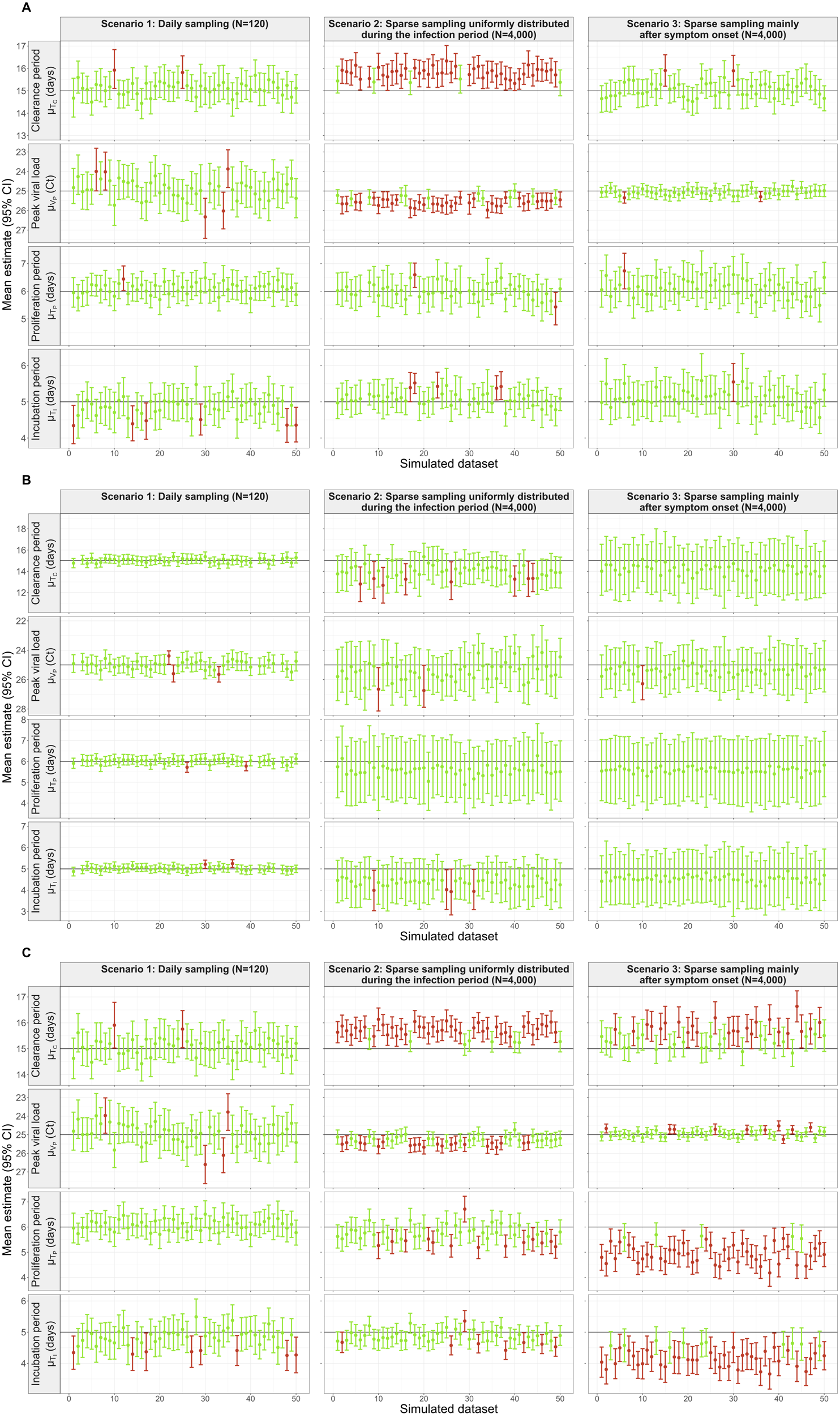

Supplement: S6 Fig — (TIFF) [file pcbi.1013811.s008.tiff]

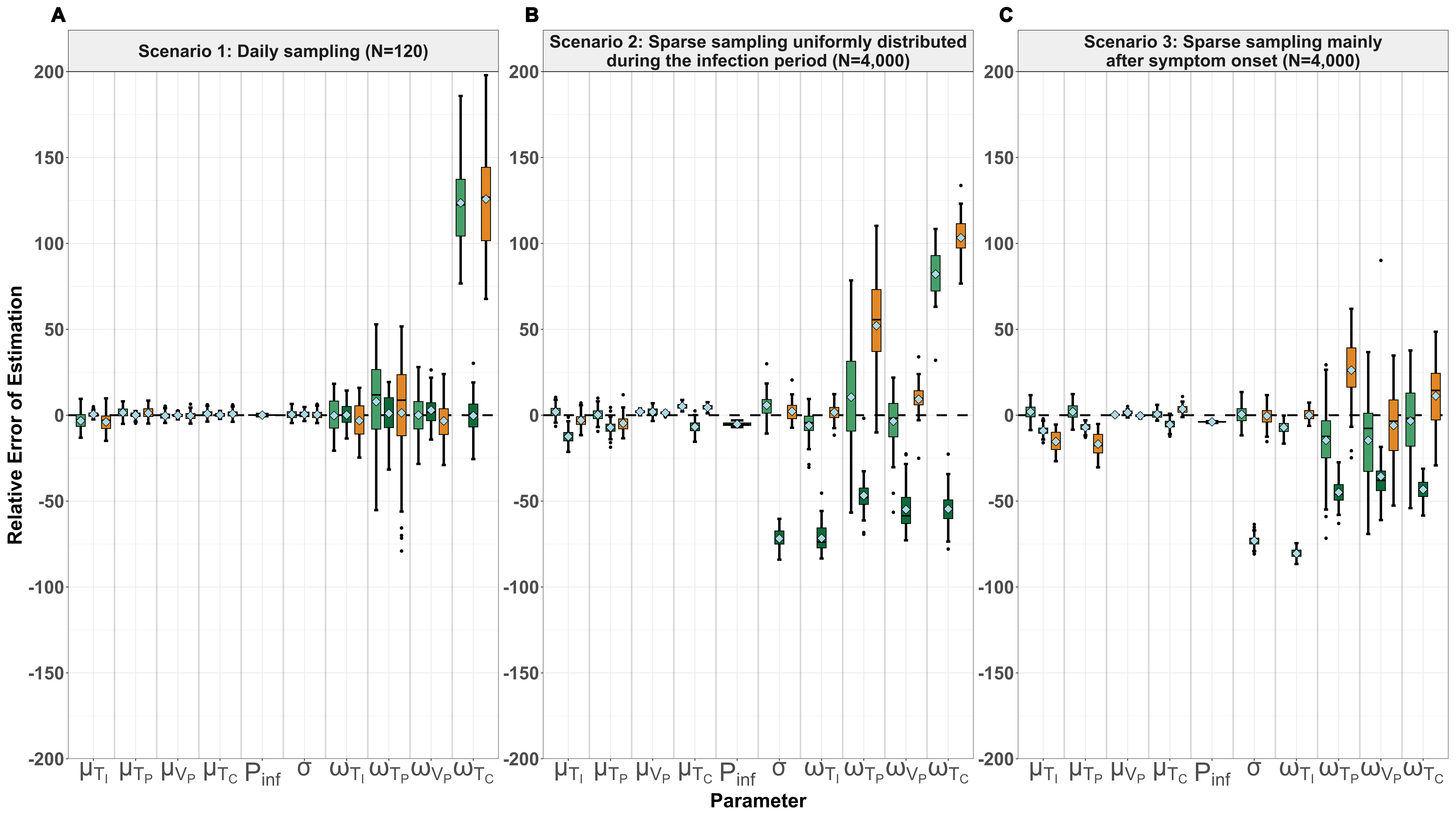

Supplement: S7 Fig — (TIFF) [file pcbi.1013811.s009.tiff]

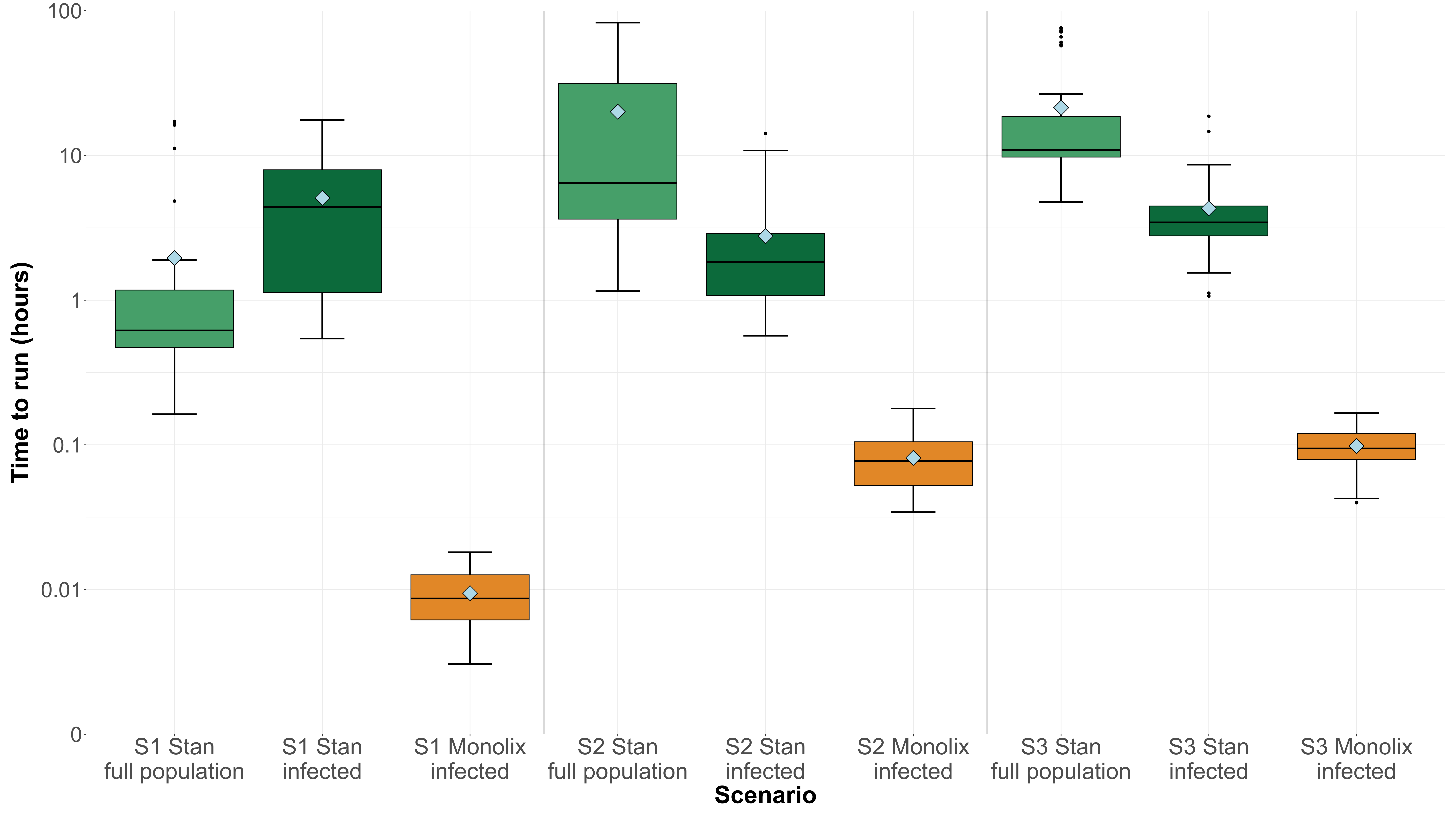

Supplement: S8 Fig — (TIFF) [file pcbi.1013811.s010.tiff]

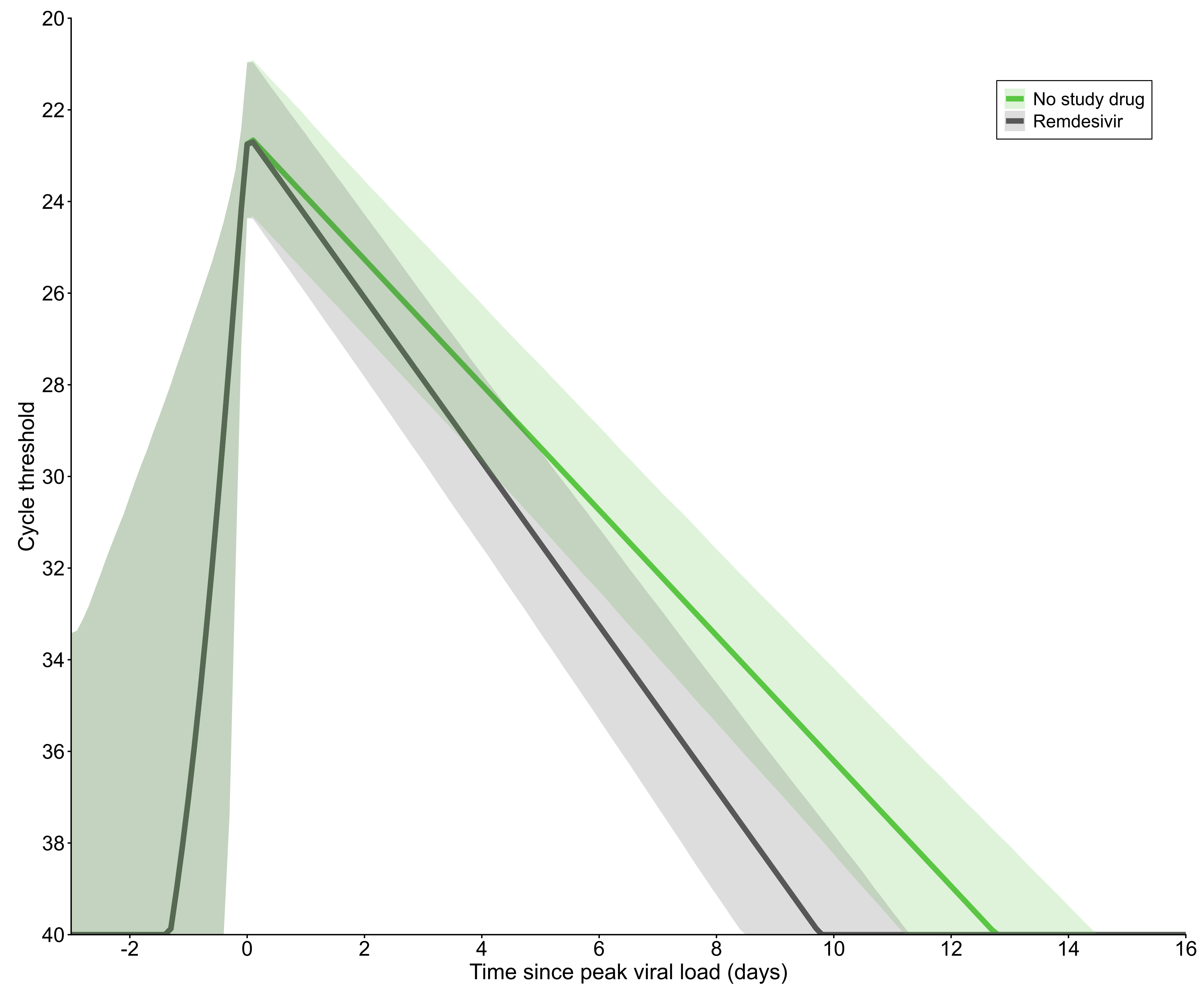

Supplement: S11 Fig — (TIFF) [file pcbi.1013811.s015.tiff]

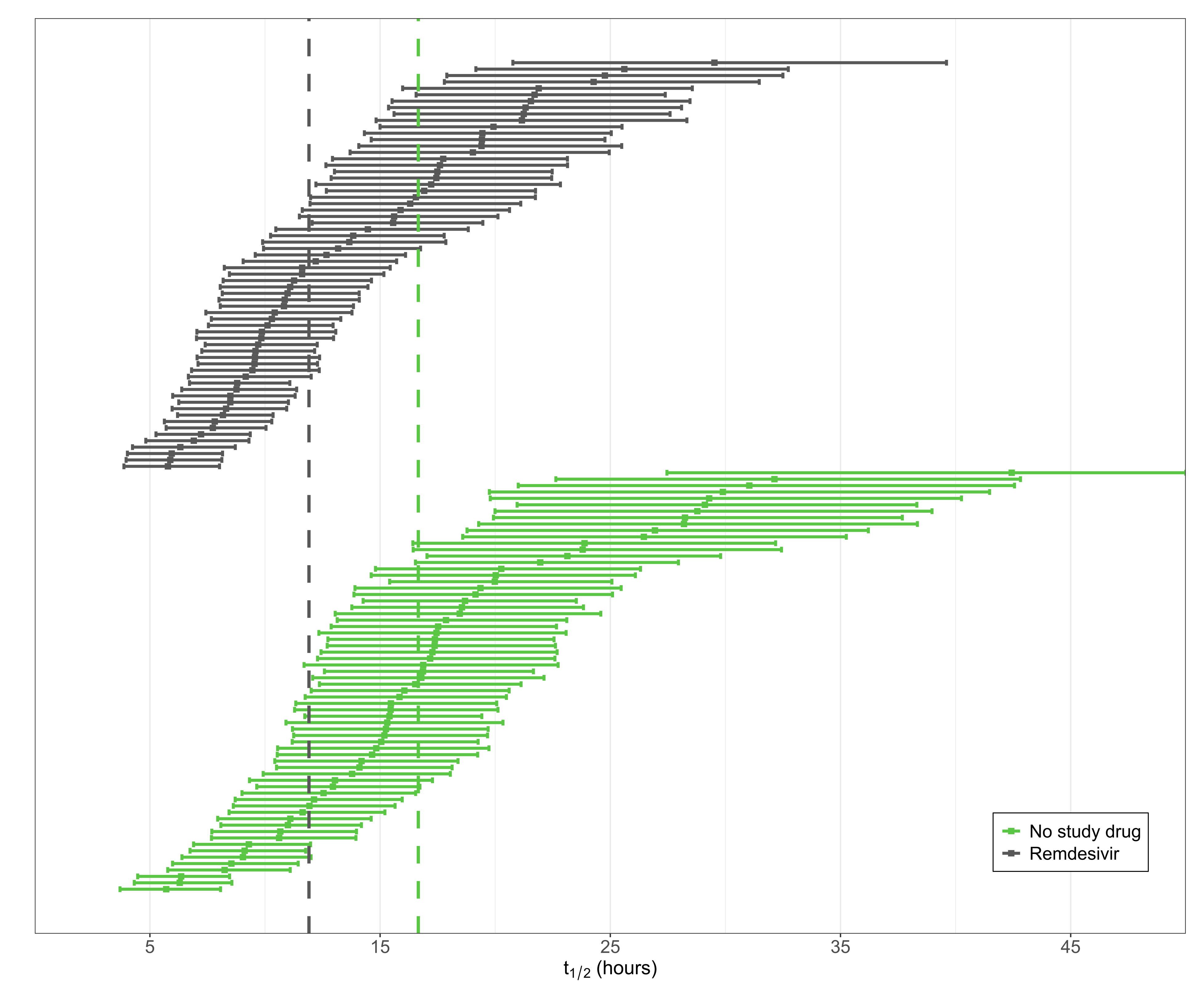

Supplement: S12 Fig — (TIFF) [file pcbi.1013811.s016.tiff]

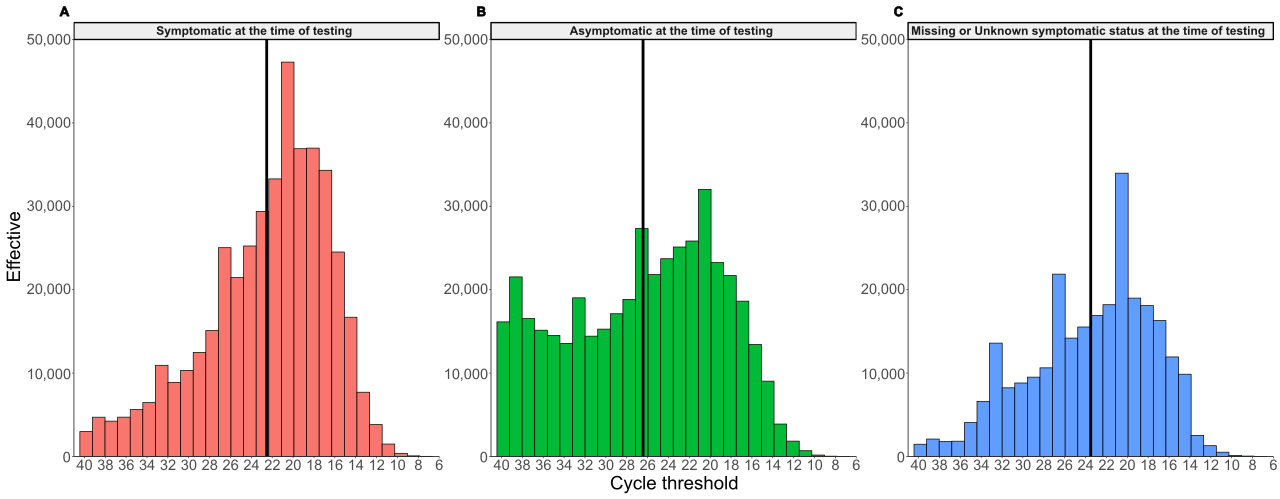

Supplement: S14 Fig — (TIFF) [file pcbi.1013811.s019.tiff]
