# Supplementary material for: Quantitative analysis of massive SARS-CoV-2 testing in the community in France in 2021–2022 reveals the associations of variant, vaccination, and age with viral dynamics in symptomatic individuals
Source: PLoS Comput Biol. 2026 Jul 27;22(7):e1013811. doi: 10.1371/journal.pcbi.1013811 (PMC13426954; doi:10.1371/journal.pcbi.1013811)
Supplement: S1 Table — (DOCX) [file pcbi.1013811.s002.docx]

## **S1 Table: Mutation and variants correspondence**

| **Mutation** | **Variant with the mutation** | **Variant without the mutation** |
| --- | --- | --- |
| E484K (A) | Beta, Gamma | Delta, BA.1, BA.2, BA.4, BA.5 |
| E484Q (B) | Kappa, Delta (2-3% of them) | Delta (98%), Alpha, Beta, Gamma, BA.1, BA.2, BA.4, BA.5 |
| L452R (C) | Delta, BA.4, BA.5 | Alpha, Beta, BA.1, BA.2 |
| Q493R* (D) | BA.1, BA.2 | Alpha, Beta, Gamma, Delta, BA.4, BA.5 |
| Del 69-70 (D) | Alpha, BA.1, BA.4, BA.5 | Beta, Gamma, Delta, BA.2 |
| K417N (D) | Beta, BA.1, BA.2, BA.4, BA.5 | Delta |
|  |  |  |

* The majority of BIOGROUP laboratories tested for the Q493R mutation
