# Supplementary material for: Quantitative analysis of massive SARS-CoV-2 testing in the community in France in 2021–2022 reveals the associations of variant, vaccination, and age with viral dynamics in symptomatic individuals
Source: PLoS Comput Biol. 2026 Jul 27;22(7):e1013811. doi: 10.1371/journal.pcbi.1013811 (PMC13426954; doi:10.1371/journal.pcbi.1013811)
Supplement: S2 Table — (DOCX) [file pcbi.1013811.s003.docx]

## **S2 Table: Rule for attributing mutation detection to the infection variant**

| **Date of the test** | **Mutation detection** | **Imputed variant of infection** |
| --- | --- | --- |
| Before 1 August 2021 | A0C0 | Pre-Omicron (likely Alpha) |
| Before 31 October 2021 | C1  A1C0 | Pre-Omicron (likely Delta)  Pre-Omicron (likely Beta) |
| After 31 October 2021 | C1 / C1D0  C0 / C0D1 | Pre-Omicron (likely Delta)  Omicron |

The letters refer to specific mutations, as listed in Table S1. The numbers denote the presence or absence of a given mutation. For each time period, we only mention the mutation profiles that were tested and detected in that period.

X1: Presence of the searched mutation; X0: Absence of the searched mutation
