# Supplementary material for: Quantitative analysis of massive SARS-CoV-2 testing in the community in France in 2021–2022 reveals the associations of variant, vaccination, and age with viral dynamics in symptomatic individuals
Source: PLoS Comput Biol. 2026 Jul 27;22(7):e1013811. doi: 10.1371/journal.pcbi.1013811 (PMC13426954; doi:10.1371/journal.pcbi.1013811)
Supplement: S3 Table — (DOCX) [file pcbi.1013811.s013.docx]

## **S3 Table:** **Estimated parameters in the model without hypothesis on viral load at the time of infection**

In this model, viral kinetics are initiated from the limit of detection (LOD) at the time of infection (t_inf_). Consequently, the incubation period (T_I_) is defined as the time between when the viral load becomes detectable and the onset of symptoms, and the proliferation period (T_P_) corresponds to the time between when the viral load becomes detectable and the peak viral load.

| **Parameter** | **Estimate (RSE in %)** | **SD of the random effect ⍵ (RSE in %)** |
| --- | --- | --- |
| $\boldsymbol{T}_{\boldsymbol{I}}$ **(days): Incubation period** |  |  |
| Pre-Omicron, vaccinated, ≥ 65 years | 2.74 (19) | 0.58 (1) |
| Pre-Omicron, vaccinated, < 65 years | 1.97 (28) |  |
| Pre-Omicron, unvaccinated, ≥ 65 years | 2.41 (60) |  |
| Pre-Omicron, unvaccinated, < 65 years | 1.93 (21) |  |
| Omicron, vaccinated, ≥ 65 years | 2.33 (33) |  |
| Omicron, vaccinated, < 65 years | 1.80 (9) |  |
| Omicron, unvaccinated, ≥ 65 years | 2.75 (29) |  |
| Omicron, unvaccinated, < 65 years | 2.13 (1) |  |
| $\boldsymbol{T}_{\boldsymbol{P}}$ **(days): Proliferation period** |  |  |
| Pre-Omicron, vaccinated, ≥ 65 years | 3.24 (22) | 0.67 (<1) |
| Pre-Omicron, vaccinated, < 65 years | 2.51 (23) |  |
| Pre-Omicron, unvaccinated, ≥ 65 years | 2.56 (124) |  |
| Pre-Omicron, unvaccinated, < 65 years | 2.34 (12) |  |
| Omicron, vaccinated, ≥ 65 years | 3.00 (21) |  |
| Omicron, vaccinated, < 65 years | 2.58 (25) |  |
| Omicron, unvaccinated, ≥ 65 years | 2.93 (86) |  |
| Omicron, unvaccinated, < 65 years | 2.70 (1) |  |
| $\boldsymbol{V}_{\boldsymbol{P}}$ **(Ct): Peak viral load** |  |  |
| Pre-Omicron, vaccinated, ≥ 65 years | 13.86 (4) | 0.02 (37) |
| Pre-Omicron, vaccinated, < 65 years | 14.69 (2) |  |
| Pre-Omicron, unvaccinated, ≥ 65 years | 13.77 (6) |  |
| Pre-Omicron, unvaccinated, < 65 years | 14.70 (2) |  |
| Omicron, vaccinated, ≥ 65 years | 15.80 (5) |  |
| Omicron, vaccinated, < 65 years | 17.09 (10) |  |
| Omicron, unvaccinated, ≥ 65 years | 15.98 (16) |  |
| Omicron, unvaccinated, < 65 years | 17.49 (<1) |  |
| $\boldsymbol{T}_{\boldsymbol{C}}$ **(days): Clearance period** |  |  |
| Pre-Omicron, vaccinated, ≥ 65 years | 19.18 (11) | 0.54 (<1) |
| Pre-Omicron, vaccinated, < 65 years | 14.79 (17) |  |
| Pre-Omicron, unvaccinated, ≥ 65 years | 22.71 (8) |  |
| Pre-Omicron, unvaccinated, < 65 years | 17.62 (8) |  |
| Omicron, vaccinated, ≥ 65 years | 16.74 (19) |  |
| Omicron, vaccinated, < 65 years | 15.17 (22) |  |
| Omicron, unvaccinated, ≥ 65 years | 21.10 (12) |  |
| Omicron, unvaccinated, < 65 years | 15.67 (<1) |  |
| $\boldsymbol{\sigma}$ **(Ct): Standard deviation of the residual error** | 3.21 (<1) | - |
