# Supplementary material for: Quantitative analysis of massive SARS-CoV-2 testing in the community in France in 2021–2022 reveals the associations of variant, vaccination, and age with viral dynamics in symptomatic individuals
Source: PLoS Comput Biol. 2026 Jul 27;22(7):e1013811. doi: 10.1371/journal.pcbi.1013811 (PMC13426954; doi:10.1371/journal.pcbi.1013811)
Supplement: S4 Table — (DOCX) [file pcbi.1013811.s014.docx]

## **S4 Table: Estimated parameters for patients in the “no study drug” and “remdesivir” arms of the PLATCOV trial**

| **Parameter** | **Estimate (RSE in %)** | **SD of the random effect ⍵ (RSE in %)** |
| --- | --- | --- |
| $\boldsymbol{T}_{\boldsymbol{I}}$ **(days): Incubation period** | 3.84 (31) | 0.27 (38) |
| $\boldsymbol{T}_{\boldsymbol{P}}$ **(days): Proliferation period** | 2.16 (69) | 0.62 (56) |
| $\boldsymbol{V}_{\boldsymbol{P}}$ **(Ct): Peak viral load** | 27.45 (3) | 0.12 (12) |
| $\boldsymbol{T}_{\boldsymbol{C}}$ **(days): Clearance period** |  |  |
| No study drug | 12.75 (7) | 0.47 (8) |
| Remdesivir | 9.73 (19) |  |
| $\boldsymbol{\sigma}$ **(Ct): Standard deviation of the residual error** | 4.14 (2) | - |
