# Supplementary material for: Quantitative analysis of massive SARS-CoV-2 testing in the community in France in 2021–2022 reveals the associations of variant, vaccination, and age with viral dynamics in symptomatic individuals
Source: PLoS Comput Biol. 2026 Jul 27;22(7):e1013811. doi: 10.1371/journal.pcbi.1013811 (PMC13426954; doi:10.1371/journal.pcbi.1013811)
Supplement: S5 Table — (DOCX) [file pcbi.1013811.s017.docx]

## **S5 Table: Age and sex characteristics by presence or absence of vaccination status information**

Data are in n (%), the number of individuals.

|  | **Individuals with a nasopharyngeal test (n = 3,879,989)** | |
| --- | --- | --- |
|  | **No information on vaccination status (n = 1,152,754)** | **Information on vaccination status  (n = 2,727,235)** |
| **Age** |  |  |
| **< 65 years** | 1,007,061 (87.4) | 2,370,295 (86.9) |
| **≥ 65 years** | 145,693 (12.6) | 356,940 (13.1) |
| **Sex** |  |  |
| **Female** | 601,056 (52.1) | 1,443,418 (52.9) |
| **Male** | 551,698 (47.9) | 1,283,817 (47.1) |
