# Supplementary material for: Quantitative analysis of massive SARS-CoV-2 testing in the community in France in 2021–2022 reveals the associations of variant, vaccination, and age with viral dynamics in symptomatic individuals
Source: PLoS Comput Biol. 2026 Jul 27;22(7):e1013811. doi: 10.1371/journal.pcbi.1013811 (PMC13426954; doi:10.1371/journal.pcbi.1013811)
Supplement: Attachment — Submitted filename: Review.docx [file pcbi.1013811.s021.docx]

**Review of “The analysis of a large dataset of PCR-test results in the community reveals the impact of age, variants and vaccination status on SARS-CoV-2 viral dynamics”**

**Summary**

This is an ambitious manuscript that seeks to improve understanding of SARS-CoV-2 viral load trajectories through analysis of large-scale community testing results. While it proposes methods that are interesting and could be valuable, the inference for these methods has severe flaws and it is unclear what the specific use would be.

**General comments**

1. This manuscript appears to address three related but distinct goals: (1) proposing, justifying, and demonstrating a statistical method for inferring viral dynamics parameters from community test results; (2) identifying the parameters for specific subpopulations for SARS-CoV-2 infection; and (3) causal inference on the effects of age, variant, and vaccination status on those parameters. I think (3) is a very challenging goal, and one that cannot be supported by the data set and methods described here, and the paper would be strengthened by focusing more clearly on (1) and (2).
   a. Causal language (“effect”, “shortens”, “impact”, “influence”) is used throughout the manuscript without addressing possible confounding, which can be particularly severe in community testing settings and with the large time dynamics that distinguish the pre-Omicron and Omicron cohorts and the unvaccinated and vaccinated cohorts.
   b. No method is developed for estimation and inference on the differences between parameters by subgroup themselves, so the causal conclusions (e.g., “strong host immunity accelerates viral clearance but does not reduce peak viral load”) are not based on specific estimation and inference results shown.
2. More generally, the representativeness (or lack thereof) of the community testing pool should be discussed more fully. While the authors discuss this to some extent (lines 358–372), more details about the reasons for testing and the reasons that some individuals had full covariates and others did not needs to be discussed in order to understand how to interpret these parameters. More details on the date of tests vs. vaccination status and age would also help in interpreting the differences in the subpopulations considered.
3. The Bayesian model simulation results are very interesting, but it is challenging to see how valuable they are when the priors selected are centered on the true simulated values. This is especially problematic when thinking about identifying the differences between subgroups. If used early in the Omicron period, for example, priors selected by an investigator would probably represent pre-Omicron values. So then it is hard to know how well this method would perform in the new setting. Additional simulations with different priors are needed to understand this fully.
4. As the authors note, the frequentist method in the simulations exhibits pretty severe undercoverage for the key parameters in realistic sampling settings. Yet the data analysis results present these intervals without much comment on this issue. The authors should provide guidance or justification on why these are still useful intervals when they do not have nominal coverage.

**Specific Comments**

1. P. 4, ll. 61–66: this, along with p. 5, l. 79, frames the main goal as identifying the effect of vaccination and variant. Please adjust if that is not the primary goal of the manuscript (see general comment 1).
2. P. 5, ll. 85–87: details on why this information was available for relatively few individuals and whether those who did have information are representative of the whole population are needed.
3. P. 5, l. 89: How is positive determined here? Is it at the 40 Ct limit of detection used later?
4. P. 6, l. 117: Normal distribution may not be appropriate for the residuals of Ct values (see, e.g., reference 1 of the manuscript). Sensitivity analysis or justification for this choice should be included.
5. P. 6, ll. 117–118: Setting this value at 50 may have a profound effect on results. Can sensitivity analyses be conducted for this?
6. P. 7, ll. 125–127: It would be useful to visualize the distribution of random effects that these models indicate, as they can be hard to understand through the numbers alone. In addition, the random effect of 0.15 seems small (especially for the periods, which can vary by much more than plus/minus 2 days). Sensitivity analysis or justification for this choice should be included.
7. P. 7, l. 136: I’m not sure what is meant by guaranteed here. I take it this means all were observed in the simulation, but there is not actually a probabilistic guarantee that would hold in general?
8. P. 7, l. 146: This should be re-numbered as Figure 2.
9. P. 8, Eqn 2: n+ and n- should be defined here rather than below equation 4. They should also be indexed by i as they may vary from person to person.
10. P. 8, ll. 165–166: Can you clarify whether P_inf_ represents a population parameter or an estimate here?
11. P. 8, l. 169: Can you clarify the specific conditional probability represented by P_TN_?
12. P. 11, Tbl. 1: It would help to add percentages of each group represented by the number in each category (e.g., female % for each column, % of individuals with 1 test, etc.)
13. P. 11, Tbl. 1: For the bottom section, it’s not clear to me how these can be per test values, but add up tot eh total number of individuals in the group. Perhaps I am missing something, but could this be clarified?
14. P. 12: It would be helpful to have figures showing the average widths of the intervals, so that their coverage performance can be assessed in light of the uncertainty they represent.
15. P. 14, l. 279: I think it is an overstatement to call this “minimal bias”; and the issues with inference should be noted here as well.
16. P. 14: I think Table S3 would be useful in the main text rather than the supplement as it is the key result of the data analysis.
17. Figure 4: The caption would be clearer if it read “percentage of PCR tests that were negative” rather than “percentage of negative tests”
18. Figure 6: can you clarify how the uncertainty on the trajectories is computed in this figure? What assumptions, if any, are made about the independence of the parameter estimates?
19. Figure S4: the scale is unclear: is it both days and Ct values?
