# Supplementary material for: Quantitative analysis of massive SARS-CoV-2 testing in the community in France in 2021–2022 reveals the associations of variant, vaccination, and age with viral dynamics in symptomatic individuals
Source: PLoS Comput Biol. 2026 Jul 27;22(7):e1013811. doi: 10.1371/journal.pcbi.1013811 (PMC13426954; doi:10.1371/journal.pcbi.1013811)
Supplement: Attachment — Submitted filename: Response_to_Reviewer.docx [file pcbi.1013811.s022.docx]

**Review:** The analysis of a large dataset of PCR-test results in the community reveals the impact of age, variants and vaccination status on SARS-CoV-2 viral dynamics

**Responses to referees and editor**

We thank the reviewers and the reviewing editor for their comments which have contributed to greatly improve the manuscript. The main changes are the following:

- We removed causal language throughout the manuscript and reframed the work as a descriptive/subgroup analysis of viral kinetics, with expanded discussion of confounding and selection biases.
- We added an external validation using PLATCOV clinical trial data, supporting the model’s ability to recover known differences in viral kinetics across groups.
- We performed additional sensitivity analyses (including alternative model assumptions and prior misspecification tests) to assess robustness of the inference framework.
- We improved transparency and reproducibility by clarifying methodology and assumptions, updating figures/tables, and making all code and models publicly available.

Please find below our point-by-point response

**Reviewer #1: James A Watson**

**Summary**

**This work uses routinely collected PCR testing data to infer the viral kinetics of SARS-CoV-2 and the effects of variant, age and sex. The authors have access to a very large dataset from a private laboratory with nearly 400K records with basic clinical data. They pair the observed CT values with the self-reported time from symptom onset to recover the population viral kinetics. This is an important demonstration of the value of these routinely collected data. I enjoyed reading the paper.**

We thank the reviewer for his positive feedback and careful critical reading of our manuscript. We have modified the analyses in line with the reviewer’s comments. We now incorporate an external validation of our modelling framework using data from the PLATCOV clinical trial, as well as additional sensitivity analyses and clarifications of key methodological assumptions. Further, we have revised the manuscript to remove causal language throughout (including title, abstract, author summary, results, and discussion) and to provide a more cautious interpretation of the findings, with an expanded discussion of potential confounding factors and limitations of the data. We have also clarified several methodological aspects, including uncertainty quantification, model assumptions, and reporting details, and made the code and models publicly available in a GitHub repository to ensure reproducibility (1). All results have been updated accordingly.

**Major points:**

1. **I think the paper would benefit from some more discussion/thinking around potential biases in the data collection. What do the authors think might be a reasonable causal structure for the data? For example, do they think it is possible that disease severity is a confounder? Suppose that older patients are generally followed-up more closely when they are sicker and therefore the observed slower clearance in older patients is in fact driven by severity rather than age? It would be useful to say what your causal assumptions are, maybe with the use of a DAG.**

We thank the reviewer for this comment and agree with this point. Interpretation of results in such observational study has to be done very cautiously. We have therefore modified the text throughout (including the title) to avoid causal language and adopt a more cautious tone. In the Discussion, we now better emphasize the potential impact of confounding factors inherent to this dataset.

Regarding your last comment, Table 1 does not suggest major differences in the number of tests across age groups.

New title:

“*Quantitative analysis of massive SARS-CoV-2 testing in the community in France in 2021-2022 reveals the associations of variant, vaccination, and age with viral dynamics in symptomatic individuals*”

Modifications made in the abstract:

*“We quantified the associations of age, vaccination status, and variant type with viral load trajectories. Age ≥65 years was consistently associated with a longer clearance time (2 to 6 days), while vaccination was associated with a shorter clearance time (2 to 4 days). Infections with Omicron variants were associated with lower peak viral load (2 to 3 Ct) and shorter clearance times (1 to 2 days) compared with pre-Omicron (Delta) infections.” Lines 30-34*

Modifications made in the author summary:

*“We found that older age was associated with longer infection duration, while vaccination was associated with a shorter duration of detectable virus without differences in peak viral levels.” Lines 47-49*

Modifications made in the Introduction section:

*“We then analysed a large dataset of community-based PCR tests collected in France in symptomatic individuals during the circulation of both Delta and Omicron variants to assess associations between variant, vaccination status, and age and viral load dynamics.” Lines 77-79*

Modifications made in the Results section:

*“The clearance period was consistently longer among individuals aged ≥65 years across variants and vaccination groups, by approximately 2 to 5 days (Figure 5), corresponding to a longer duration of detectable viral load (Figure 6). In pre-Omicron infections, the clearance period among unvaccinated individuals aged ≥65 years was 22.5 days (95% confidence interval: 21.2–23.8), compared with 17.5 days (17.3–17.7) in individuals aged <65 years. A similar pattern was observed for Omicron infections, with clearance of 21.1 days (19.7–22.6) in unvaccinated individuals aged ≥65 years versus 15.9 days (15.7–16.2) in individuals aged <65 years. Differences in peak viral load by age were more limited, with lower Ct values (reflecting higher viral load) in individuals aged ≥65 years, e.g. 16.0 (15.6–16.5) versus 17.6 (17.5–17.7) for Omicron infections in unvaccinated individuals aged ≥65 years versus <65 years, respectively.*

*Shorter clearance periods were observed among vaccinated individuals across variants and age groups, by approximately 2 to 4 days (Figure 5), corresponding to a shorter duration of detectable viral load (Figure 6). In pre-Omicron infections, clearance time was 22.5 days (21.2–23.8) in unvaccinated versus 18.9 days (18.1–19.7) in individuals aged ≥65 years, and 17.5 days (17.3–17.7) versus 14.7 days (14.5–14.9) in vaccinated individuals aged <65 years. In Omicron infections, differences by vaccination status were smaller among individuals aged <65 years, with clearance times of 15.9 days (15.7-16.2) in unvaccinated, and 15.4 days (15.3–15.5) in vaccinated individuals. Larger differences were observed among individuals aged ≥65 years, with clearance times of 21.1 days (19.7-22.6) in unvaccinated and 16.9 days (16.6-17.2) in vaccinated individuals, corresponding to a difference of 4.2 days. Peak viral load was similar across vaccination groups, with differences consistently <0.5 cycle threshold.*

*Omicron infections were consistently associated with lower peak viral load across vaccination and age groups, by approximately 2 to 3 Ct (Figure 5) compared with pre-Omicron variants. Pre-Omicron infections showed Ct values around 14–15, e.g. 14.8(14.7–14.9) in unvaccinated individuals aged <65 years, compared with values around 17–18 in Omicron infections, e.g. 17.6 (17.5–17.7) in the same age group. Clearance period also tended to be shorter in Omicron compared with pre-Omicron infections, with the largest difference observed among vaccinated individuals aged ≥65 years, with a duration of 18.9 days (18.1-19.7) in pre-Omicron infections and 16.9 days (16.6-17.2) in Omicron infections.”
Lines 288-315*

Modifications made in the Discussion section:

*“We showed by simulation that key patterns of viral kinetics can be estimated from community-based testing data with good accuracy and precision. The modelling framework was subsequently applied to a large dataset of PCR results collected in French community laboratories between July 2021 and March 2022, and showed that age >65 years was associated with differences in viral kinetics parameters, including a longer duration of infection of 2 to 6 days. Vaccination was associated with shorter clearance periods across variants and age groups, by 2 to 4 days, but was not associated with differences in peak viral load. Infections with Omicron variants were associated with lower peak viral load across vaccination and age groups, by approximately 2 to 3 Ct compared with Pre-Omicron variants. Clearance times also tended to be shorter in Omicron compared with pre-Omicron infections, with differences of up to 2 days.” Lines 318-327*

*“Moreover, it is the first, using community-based data, to robustly characterize the independent associations of Omicron and vaccination on within-host viral dynamics.” Lines 334-336*

Additions to the Discussion section:

*“Beyond methodological issues, the main limitation of these data is that they were not collected for research purposes and may be associated with various limiting or confounding factors. For instance, the (self-reported) occurrence or the period of symptom onset, which is used to reconstruct viral trajectories, is prone to recall bias (Figure S9)(33). This uncertainty was not accounted for in our model and the data of symptom onset was imputed at the median of declared interval of symptom onset. Although most individuals declared an interval for symptom onset of one day, a Bayesian inference framework could naturally incorporate this uncertainty in future work. Although our selected population did not appear to differ from the overall dataset based on observed characteristics, restricting the analysis to individuals with complete information, in particular symptom and vaccination status,* *may have induced selection bias (Table S5). More generally, data were collected during a period where the vast majority of PCR tests were fully reimbursed by the national health insurance (34), limiting financial barriers to testing. Nevertheless, the motivation for undergoing testing have evolved over time, particularly in the context of the French “passe sanitaire” implemented during the study period, which may have resulted in differential testing behaviour between vaccinated and unvaccinated individuals. This policy may also partly explain the high prevalence of unvaccinated individuals in our dataset (Figure S13). Moreover, several sources of residual confounding may persist. First, disease severity could act as an unmeasured confounder, as information on symptom type and intensity was not available in our dataset. Similarly, no clinical data were available, while presence of risk factors (eg, chronic disease) may impact both the use of tests and viral load. In addition, information on the time since last vaccine dose was rarely recorded, preventing us from accounting for potential waning immunity effects. Furthermore, during the study period, reimbursed rapid antigen tests were widely available in pharmacies, raising the possibility that individuals seeking PCR testing may not be representative of the general population.” Lines 357-379*

2. **Validation on an external dataset. For this research to be fully convincing, I would ideally hope to see the inferred parameters validated using an external dataset where patients have been prospectively followed. Ideally this would a dataset such as the US NBA (https://journals.plos.org/plosbiology/article?id=10.1371/journal.pbio.3001333), but if there is no good equivalent for this timeframe then just a prospective study in symptomatic patients (eg PLATCOV studies)? If the authors could show that their inferred parameters approximate well parameters inferred from more detailed data, then this would give a much stronger result. This is especially important as the authors don’t use the best method (fully Bayesian) because of the computational issues. It’s clear from the simulations that the Monolix method gives biased inference.**

We thank the reviewer for this excellent suggestion. External validation of the model would provide additional support for its generalizability to datasets from different contexts, beyond demonstrating its ability to estimate covariate effects on model parameters. In addition, it would ensure reproducibility of our methods, as we could not share the data used in this article (see below). We have therefore included an additional analysis using an external dataset in the Supplementary Materials and have discussed these results in the Discussion section.

We decided to use the PLATCOV data and not the US NBA dataset as the latter does not have symptom onset, and therefore would have required changes in the method.

We modelled Ct values from the “no study drug” and “remdesivir” arms (2) using Monolix to assess the acceleration of viral clearance in patients treated with remdesivir. We tested a treatment effect on the clearance phase, assuming that individuals in both treatment arms share similar incubation (T_I_), proliferation (T_P_), and peak viral load (V_P_) parameters. Our estimates suggest a difference in clearance duration between treatment arms, with an estimated reduction of approximately three days in the remdesivir arm (9.73 days vs 12.75 days; Wald test, P = 2.7 × 10⁻⁷) (see Table S4 and Figure S11-S12, added in the appendix).

To compare the analysis with the original study, we computed viral clearance half-lives for each patient (Figure S11). Results are consistent with those reported by the PLATCOV group, with median estimated viral clearance half-lives of 11.9 hours (range, 5.8–29.5) in the remdesivir arm and 16.6 hours (range, 5.7–42.4) in the control arm, compared with 12.8 hours (4.8–50.0) and 18.0 hours (3.6–46.7) in the remdesivir and control arms, respectively, in the original study. Altogether, these findings support the generalizability of our model and its ability to identify differences in viral kinetics across subgroups.

The scripts and models have been added to the GitHub repository (1).

Additions to the Discussion section:

*“The model can also be used in other settings with richer dataset or to assess differences across treatment groups in clinical trials. For instance, we performed an external validation using data from the PLATCOV trial (32), comparing viral load in untreated vs remdesivir-treated individuals. Consistent with previously published results (32), the model identified a significantly shorter clearance phase in the remdesivir arm, with estimated viral clearance half-lives consistent with those reported in the original study (Table S4, Figures S11–S12).” Lines 350-356*

**Table S4: Estimated parameters for patients in the “no study drug” and “remdesivir” arms of the PLATCOV trial**

| **Parameter** | **Estimate (RSE in %)** | **SD of the random effect ⍵ (RSE in %)** |
| --- | --- | --- |
| $\boldsymbol{T}_{\boldsymbol{I}}$ **(days): Incubation period** | 3.84 (31) | 0.27 (38) |
| $\boldsymbol{T}_{\boldsymbol{P}}$ **(days): Proliferation period** | 2.16 (69) | 0.62 (56) |
| $\boldsymbol{V}_{\boldsymbol{P}}$ **(Ct): Peak viral load** | 27.45 (3) | 0.12 (12) |
| $\boldsymbol{T}_{\boldsymbol{C}}$ **(days): Clearance period** |  |  |
| No study group | 12.75 (7) | 0.47 (8) |
| Remdesivir | 9.73 (19) |  |
| $\boldsymbol{\sigma}$ **(Ct): Standard deviation of the residual error** | 4.14 (2) | - |

**Figure S11: Distribution of cycle threshold values according to symptomatic status declared at the time of testing**

Model-based kinetics of the viral load value (in Ct) in the two treatment arms (green: no study drug; black: remdesivir). Lines show the mean predicted Ct trajectory, with shaded areas indicating the 95% confidence interval.

**Figure S12: Individual estimate of the viral clearance half-lives**

Individual estimate of the viral clearance half-lives with point estimates (squares) and 80% confidence intervals (lines). The vertical dashed lines show the median population viral clearance half-lives by treatment arm (lower: no study drug; upper: remdesivir). The median estimated viral clearance half-lives were 11.9 (range, 5.8-29.5) hours in the remdesivir arm and 16.6 (range, 5.7-42.4) hours in the control arm, compared to 12.8 (4.8-50.0) hours and 18.0 (3.6-46.7) hours in the remdesivir and control arm respectively in the original article (2).

3. **Are the parameters inferred believable? This links to the point above and may reflect my misunderstanding. In table S3, the peak viral load estimates are between 14 and 17 on the CT scale. This seems very low (ie very high viral load)? Given that this is the population average by subgroup, I find the values hard to believe. The prior is 25 which seems more reasonable. Very few CT values in Fig S8 are below 14. Does this represent an issue in the inference?**

We thank the reviewer for this interesting question. First, our simulation study shows that the model in Scenario 3 (similar to the analysed dataset) can accurately infer the peak viral load ($\mu_{V_{P}}$). It is correct that the histogram of Ct value distributions (Figure S14A) shows that only a small proportion of values are below 17 ; however, most of these data correspond to Ct values collected >2 days after symptom onset, i.e., after the peak viral load and in the descending phase.

Ct values are highly dependent on the PCR technique used (3), and can reach values below 20 Ct (4–7). Finally, in our analysis, we do not comment on the absolute value of the estimated peak but only on the differences in estimated values between groups.

4. **No code or data. Will the authors make the code available? And can they make the CT data available as well? Given that this is a computational journal, code/data availability is very important for reproducibility.**

We created a GitHub repository (1), including scripts to simulate the data, an example of a simulated dataset, the Monolix and Stan models used (along with scripts to run them), as well as the scripts used for the external validation of our model on the PLATCOV dataset.

Added Data availability statement section:

*“For regulatory and ethical reasons, Biogroup does not wish to release the data publicly. Indeed, under French regulations, patients have the right to object to the use of their data, as well as the right to review and be notified about the nature of the data use and the research project. Open access to the data would not meet the need for traceability of the data and analyses. However, researchers can contact Biogroup to obtain the dataset at* [*gina.cosentino@biogroup.fr*](mailto:gina.cosentino@biogroup.fr)*.” Lines 402-406*

**Minor points:**

5. **Have the authors thought about using this approach for other viral infections? Notably ‘flu**

We thank you for this insightful comment. Indeed, the pipeline developed in this work could be adapted and applied to similar virological data from other acute infections. Currently, modelling studies of viral dynamics based on community data from multiplex (triplex) testing (SARS-CoV-2, influenza, RSV) collected during the 2024–2025 and 2025–2026 winter seasons are ongoing and have not yet been published.

**Equation 2 notation is confusing as f is used for the model structure and the normal pdf. Could just use another letter for the model, e.g. g(x)?**

We thank you for pointing this out, the structural model function has been renamed g(x) to avoid confusion.

6. **Is there any temporal trend in clearance values not explained by variant? Eg as suggested by the PLATCOV study (https://pubmed.ncbi.nlm.nih.gov/38677300/). This could be looked at by comparing model random effect estimates with calendar date**.

We thank the reviewer for this insightful comment. Indeed, other factors not captured by age, vaccination status, and infecting variant, such as the number of vaccine doses or the time since the last dose (which are correlated with the testing date), may influence the peak viral load and the viral clearance duration. We examined the distributions of individual random effects by week of testing for both the viral load peak ($\eta_{Vp}$) and the viral clearance period ($\eta_{Tc}$), and observed no temporal trends in our data across weeks (Figure S15).

Additions to the Discussion section:

*“*Finally, we also investigated potential differences according to temporal trends, irrespective of circulating variant or immune background (8)*. To explore this, we examined the estimated random effects of peak viral load and viral clearance duration according to the date of testing (Figure S15), but did not observe any temporal pattern.” Lines 387-390*

**Figure S15: Distribution of the estimated individual random effects of peak viral load and clearance period according to the date of testing**

Distribution of estimated individual random effects of peak viral load $\mu_{Vp}\times e^{\eta_{Vp}}$ (left) and clearance period $\mu_{Tc}\times e^{\eta_{Tc}}$ (right) according to the week of testing. The central line in each box indicates the median, the box represents the interquartile range (IQR; 25%–75%), and the whiskers extend to 1.5 × IQR or to the most extreme values.

**Reviewer #2:**

**Summary**

**This is an ambitious manuscript that seeks to improve understanding of SARS-CoV-2 viral load trajectories through analysis of large-scale community testing results. While it proposes methods that are interesting and could be valuable, the inference for these methods has severe flaws and it is unclear what the specific use would be.**

We thank the reviewer for the detailed and constructive comments, which have substantially improved the manuscript. In response, we have removed causal language throughout (title, abstract, main text, and discussion). We have expanded the discussion of potential confounding and selection biases in community testing data, and clarified the representativeness of the study population. We have also strengthened the methodological framework by improving the description of uncertainty quantification and model assumptions, and by adding additional sensitivity analyses, including further simulations assessing prior sensitivity. All analyses and results have been updated accordingly.

**Major points:**

7. **This manuscript appears to address three related but distinct goals: (1) proposing, justifying, and demonstrating a statistical method for inferring viral dynamics parameters from community test results; (2) identifying the parameters for specific subpopulations for SARS-CoV-2 infection; and (3) causal inference on the effects of age, variant, and vaccination status on those parameters. I think (3) is a very challenging goal, and one that cannot be supported by the data set and methods described here, and the paper would be strengthened by focusing more clearly on (1) and (2).**
   7.1. **Causal language (“effect”, “shortens”, “impact”, “influence”) is used throughout the manuscript without addressing possible confounding, which can be particularly severe in community testing settings and with the large time dynamics that distinguish the pre-Omicron and Omicron cohorts and the unvaccinated and vaccinated cohorts.**

We thank the reviewer for this very insightful comment and agree that the data we rely on do not support a causal interpretation. We have revised the title, abstract, author summary, results, and discussion to remove causal language. In addition, we have expanded the discussion of potential confounding factors in the Discussion section. These modifications and additions are detailed in our response to comment number 1.

7.2. **No method is developed for estimation and inference on the differences between parameters by subgroup themselves, so the causal conclusions (e.g., “strong host immunity accelerates viral clearance but does not reduce peak viral load”) are not based on specific estimation and inference results shown.**

We thank the reviewer for this comment. Following the previous comment we agree that causal interpretation should be done very cautiously and wording has been changed to be more precise. Given the presence of potential confounding factors and the size of the dataset, we decided to analyse viral load in each subgroup, and to only compare mean parameter values across groups, without testing for a statistically significant difference.

Regarding the ability of the model to estimate differences, and following comments from Reviewer 1, we now include an external validation of the model using data from the PLATCOV clinical trial (see our response to comment number 2). In this analysis, we identified a significant difference in viral clearance duration between untreated and treated individuals, with estimates very close to those reported in the original study. These results show that our model can be used to identify differences in viral kinetic parameters across groups.

8. **More generally, the representativeness (or lack thereof) of the community testing pool should be discussed more fully. While the authors discuss this to some extent (lines 358–372), more details about the reasons for testing and the reasons that some individuals had full covariates and others did not need to be discussed in order to understand how to interpret these parameters. More details on the date of tests vs. vaccination status and age would also help in interpreting the differences in the subpopulations considered.**

We agree with this comment, as the reasons for testing may differ across subgroups. For instance, unvaccinated individuals were required to take a recent negative test to access specific places (eg, restaurants) during parts of the study period.

Thus, it is not surprising to find that the proportion of unvaccinated individuals in the dataset (infected and not infected) is larger than in the French population during the same period of time (Figure S12). Regarding the question on missing data, we also looked at whether individuals without vaccination status data differed from those with available information (Table S5). We found that these groups were comparable in terms of sex and age.

These points have now been added to the Supplementary Materials and discussed in the Discussion section.

Additions to the Discussion section:

*“Although our selected population did not seem based on data collected, it is also possible that the restriction of our analysis to individuals with complete information, in particular symptom and vaccination status, may induce a selection bias (Table S5). More generally, data were collected during a period where the vast majority of PCR tests were fully reimbursed by the national health insurance (34), limiting financial barriers to testing. Nevertheless, the motivation for undergoing testing have evolved over time, particularly in the context of the French “passe sanitaire” implemented during the study period, which may have resulted in differential testing behaviour between vaccinated and unvaccinated individuals. This policy may also partly explain the high prevalence of unvaccinated individuals in our dataset (Figure S13).“ Lines 362-372*

**Table S5: Age and sex characteristics by presence or absence of vaccination status information**

Data are in n (%), the number of individuals.

|  | **Individuals with a nasopharyngeal test (n = 3,879,989)** | |
| --- | --- | --- |
|  | **No information on vaccination status (n = 1,152,754)** | **Information on vaccination status  (n = 2,727,235)** |
| **Age** |  |  |
| **< 65 years** | 1,007,061 (87.4) | 2,370,295 (86.9) |
| **≥ 65 years** | 145,693 (12.6) | 356,940 (13.1) |
| **Sex** |  |  |
| **Female** | 601,056 (52.1) | 1,443,418 (52.9) |
| **Male** | 551,698 (47.9) | 1,283,817 (47.1) |

**Figure S13: Proportion of vaccinated individuals by week of testing by age category**

Proportion of vaccinated individuals by testing week and age category among all nasopharyngeal tests (N = 5,915,857). Orange: proportion vaccinated; grey: proportion unvaccinated. Solid green line: temporal evolution of national vaccination coverage by age group across weeks (9).

9. **The Bayesian model simulation results are very interesting, but it is challenging to see how valuable they are when the priors selected are centered on the true simulated values. This is especially problematic when thinking about identifying the differences between subgroups. If used early in the Omicron period, for example, priors selected by an investigator would probably represent pre-Omicron values. So then it is hard to know how well this method would perform in the new setting. Additional simulations with different priors are needed to understand this fully.**

We thank the reviewer for this relevant question regarding the extent to which prior distributions influence the posterior distribution. To assess the potential impact of the chosen prior distributions for the peak viral load and viral clearance parameters, we performed additional analyses on a randomly selected dataset from Scenario 3 (which mimics the community database we analysed) using several misspecifications of the prior distributions while keeping a relatively large variance, within the two Bayesian statistical approaches developed in our study. Specifically, we explored situations where the true values of the peak viral load and the clearance period were 25 Ct and 15 days, respectively:

- One analysis with prior of $\mu_{Vp}$ ~ N(20, 2) (−20%), with the other priors unchanged
- One analysis with prior of $\mu_{Vp}$~ N(30, 2) (+20%), with the other priors unchanged
- One analysis with prior of $\mu_{Tc}$~ N(12, 2) (−20%), with the other priors unchanged
- One analysis with prior of $\mu_{Tc}$~ N(18, 2) (+20%), with the other priors unchanged

These points have now been added to the Supplementary Materials and discussed in the Discussion section.

Additions to the Discussion section:

*“In addition, Bayesian approaches can be sensitive to prior specifications; however, in our case, posterior distributions appeared only weakly influenced by the priors (Figure S10).” Lines 342-343*

**Figure S10: Sensitivity to prior specification**

Sensitivity of the Bayesian inference framework to prior specification in Scenario 3 (community dataset design). Panel A shows results when modelling the entire population, and Panel B shows results when modelling only individuals with at least one positive PCR test.

The model including the entire population is in broad terms not sensitive to prior misspecification of ±20% for the peak viral load and viral clearance parameters (Panel A).

In the model including only individuals with at least one positive PCR test (thus including less information on the population), we observe only a small sensitivity and when using a prior for the peak viral load with a mean 20% lower than the simulated value, leading to a slight underestimation of peak viral load; and when using a prior for the viral clearance period with a mean 20% lower than the simulated value, leading to a slight underestimation of the viral clearance (Panel B).

10. **As the authors note, the frequentist method in the simulations exhibits pretty severe undercoverage for the key parameters in realistic sampling settings. Yet the data analysis results present these intervals without much comment on this issue. The authors should provide guidance or justification on why these are still useful intervals when they do not have nominal coverage.**

We thank the reviewer for this comment. The reduced coverage of the clearance time in Scenario 3 when using Monolix arises from an underestimation of the standard error. To illustrate this, we have added figures in the Supplementary Materials showing the 95% credibility and confidence intervals of the estimated parameters across each simulated dataset, inference method, and scenario (**Figure S6**).

We believe that this lower coverage of the clearance duration with Monolix is not a major concern, given the relatively large effect sizes observed between subgroups in the community data analysis, which range from 2 to 5 days.

Modifications made in the Results section:

*“The coverage rate was much better when using a Bayesian framework, and a tendency of Monolix to underestimate the standard errors of parameter estimates (Figure S6C).” Lines 246-247*

**Figure S6: Estimates and associated uncertainty of population parameters**

Estimates and corresponding 95% confidence or credible intervals of population parameters from the simulation study, for each dataset, scenario, and modelling approach. Panel A: modelling of the full population using Stan; Panel B: modelling of individuals with at least one positive PCR test using Stan; Panel C: modelling of individuals with at least one positive PCR test using Monolix. The solid horizontal line represents the true simulated value. Green indicates that the confidence (or credible) interval contains the true simulated value, whereas red indicates that it does not.

**Minor points:**

11. **P. 4, ll. 61–66: this, along with p. 5, l. 79, frames the main goal as identifying the effect of vaccination and variant. Please adjust if that is not the primary goal of the manuscript (see general comment 1).**

Following the revision of our objectives and results, as well as the expansion of the discussion on potential biases in our data (see comment number 1), we believe that one of the main objectives can be stated as identifying differences between subpopulations defined by age, vaccination status, and infecting variant.

12. **P. 5, ll. 85–87: details on why this information was available for relatively few individuals and whether those who did have information are representative of the whole population are needed.**

The data analysed originate from routine clinical practice and were not collected under a predefined study protocol, resulting in a substantial amount of missing data. We believe that our approach to imputing missing information on variant, vaccination status, and time since symptom onset helps limit potential inclusion bias. More specifically, age and sex are almost systematically recorded (Figure S9). The variant of infection could be determined in the majority of cases using sequencing data, screening results, and national epidemiological trends. Most of the remaining missing data correspond to tests performed in December 2021, a period during which Delta and Omicron were co-circulating, although neither exceeded 95% prevalence. We therefore believe that these missing data are unlikely to introduce substantial bias when comparing viral kinetics across subgroups. Individuals with missing vaccination status do not appear to differ from those with available information (see comment number 8, Table S5). Finally, restricting the analysis to symptomatic individuals introduces a selection bias, and we have revised the title to better reflect the population analysed in our study (see comment number 1).

13. **P. 5, l. 89: How is positive determined here? Is it at the 40 Ct limit of detection used later?**

We thank the reviewer for this comment. The sentence has been simplified to remove any ambiguity.

Modifications made in the Materials and Methods section:

*“We considered an individual as infected if at least one PCR test with quantified Ct value was available.” Lines 89-90*

14. **P. 6, l. 117: Normal distribution may not be appropriate for the residuals of Ct values (see, e.g., reference 1 of the manuscript). Sensitivity analysis or justification for this choice should be included.**

We thank the reviewer for this comment. We agree that a Gaussian additive error model may not be the most appropriate choice to represent measurement error in Ct values, which could increase with higher PCR cycle numbers. However, the residual error term (σ) in our model does not only reflect measurement error in Ct values, but also accounts for model misspecification relative to the observed viral kinetics. In this context, there is no clear indication that a Gaussian additive error is inappropriate.

Nevertheless, we performed an alternative analysis using a proportional error model instead of an additive one, and examined the observations versus predictions (see below). The model with proportional error did not provide a better fit to the data (Figure A) compared with the additive error model (Figure B).

**Figure A : Observations versus predictions for the model with proportional error**

Dots represent observations versus predictions; the red line indicates the identity line (observed = predicted).

**Figure A : Observations versus predictions for the model with additive error**

Dots represent observations versus predictions; the red line indicates the identity line (observed = predicted).

15. **P. 6, ll. 117–118: Setting this value at 50 may have a profound effect on results. Can sensitivity analyses be conducted for this?**

We thank the reviewer for this interesting remark, and we developed an alternative model that does not assume a viral load of 50 Ct at the time of infection (V_inf_ = 50 Ct). In this model, viral kinetics are initiated from the limit of detection (LOD) at the time of infection (t_inf_). Consequently, the incubation period (T_I_) is defined as the time between when the viral load becomes detectable and the onset of symptoms, and the proliferation period (T_P_) corresponds to the time between when the viral load becomes detectable and the peak viral load. As a result, both periods are shorter than those estimated with the model assuming an initial viral load of V_inf_ = 50 Ct, while the peak viral load (V_P_) and the clearance duration (T_C_) are similar to those obtained with the model used in the main analysis. An additional section has been included in the Supplementary Materials, and this sensitivity analysis is now discussed in the Discussion section.

Additions to the Discussion section:

*“Our model assumes a Ct value of 50 at the time of infection. To assess the robustness of this assumption, we conducted a sensitivity analysis using an alternative model that did not rely on it. The results were consistent with those of the main analysis (Table S3).” Lines 348-350*

**Table S3:** **Estimated parameters in the model without hypothesis on viral load at the time of infection**

In this model, viral kinetics are initiated from the limit of detection (LOD) at the time of infection (t_inf_). Consequently, the incubation period (T_I_) is defined as the time between when the viral load becomes detectable and the onset of symptoms, and the proliferation period (T_P_) corresponds to the time between when the viral load becomes detectable and the peak viral load.

| **Parameter** | **Estimate (RSE in %)** | **SD of the random effect ⍵ (RSE in %)** |
| --- | --- | --- |
| $\boldsymbol{T}_{\boldsymbol{I}}$ **(days): Incubation period** |  |  |
| Pre-Omicron, vaccinated, ≥ 65 years | 2.74 (19) | 0.58 (1) |
| Pre-Omicron, vaccinated, < 65 years | 1.97 (28) |  |
| Pre-Omicron, unvaccinated, ≥ 65 years | 2.41 (60) |  |
| Pre-Omicron, unvaccinated, < 65 years | 1.93 (21) |  |
| Omicron, vaccinated, ≥ 65 years | 2.33 (33) |  |
| Omicron, vaccinated, < 65 years | 1.80 (9) |  |
| Omicron, unvaccinated, ≥ 65 years | 2.75 (29) |  |
| Omicron, unvaccinated, < 65 years | 2.13 (1) |  |
| $\boldsymbol{T}_{\boldsymbol{P}}$ **(days): Proliferation period** |  |  |
| Pre-Omicron, vaccinated, ≥ 65 years | 3.24 (22) | 0.67 (<1) |
| Pre-Omicron, vaccinated, < 65 years | 2.51 (23) |  |
| Pre-Omicron, unvaccinated, ≥ 65 years | 2.56 (124) |  |
| Pre-Omicron, unvaccinated, < 65 years | 2.34 (12) |  |
| Omicron, vaccinated, ≥ 65 years | 3.00 (21) |  |
| Omicron, vaccinated, < 65 years | 2.58 (25) |  |
| Omicron, unvaccinated, ≥ 65 years | 2.93 (86) |  |
| Omicron, unvaccinated, < 65 years | 2.70 (1) |  |
| $\boldsymbol{V}_{\boldsymbol{P}}$ **(Ct): Peak viral load** |  |  |
| Pre-Omicron, vaccinated, ≥ 65 years | 13.86 (4) | 0.02 (37) |
| Pre-Omicron, vaccinated, < 65 years | 14.69 (2) |  |
| Pre-Omicron, unvaccinated, ≥ 65 years | 13.77 (6) |  |
| Pre-Omicron, unvaccinated, < 65 years | 14.70 (2) |  |
| Omicron, vaccinated, ≥ 65 years | 15.80 (5) |  |
| Omicron, vaccinated, < 65 years | 17.09 (10) |  |
| Omicron, unvaccinated, ≥ 65 years | 15.98 (16) |  |
| Omicron, unvaccinated, < 65 years | 17.49 (<1) |  |
| $\boldsymbol{T}_{\boldsymbol{C}}$ **(days): Clearance period** |  |  |
| Pre-Omicron, vaccinated, ≥ 65 years | 19.18 (11) | 0.54 (<1) |
| Pre-Omicron, vaccinated, < 65 years | 14.79 (17) |  |
| Pre-Omicron, unvaccinated, ≥ 65 years | 22.71 (8) |  |
| Pre-Omicron, unvaccinated, < 65 years | 17.62 (8) |  |
| Omicron, vaccinated, ≥ 65 years | 16.74 (19) |  |
| Omicron, vaccinated, < 65 years | 15.17 (22) |  |
| Omicron, unvaccinated, ≥ 65 years | 21.10 (12) |  |
| Omicron, unvaccinated, < 65 years | 15.67 (<1) |  |
| $\boldsymbol{\sigma}$ **(Ct): Standard deviation of the residual error** | 3.21 (<1) | - |

16. **P. 7, ll. 125–127: It would be useful to visualize the distribution of random effects that these models indicate, as they can be hard to understand through the numbers alone. In addition, the random effect of 0.15 seems small (especially for the periods, which can vary by much more than plus/minus 2 days). Sensitivity analysis or justification for this choice should be included.**

We agree with the reviewer that a representation of the simulated individual parameters was missing. We have now added this in the Supplementary Materials (Figure S2). Although the choice of a random effect of 0.15 may appear somewhat small, we believe it adequately reflects the observed variability of the parameters (10–13).

Regarding the incubation period, it has been estimated to range between 3.5 and 5 days for the Delta and Omicron variants, with a variability of approximately two days (10). For the proliferation period and peak viral load, the simulated variability is slightly higher than that observed in empirical data, which appears to be relatively low (11,12). For viral clearance, a random effect of 0.15 corresponds to a variability of approximately ±3 days; this choice was informed by estimates reported in human challenge studies (13).

**Figure S2:** **Simulated individual parameters from the simulation study**

Simulated individual parameters in the simulation study. Solid vertical lines: mean of the distribution; dashed vertical lines: 5^th^ and 95^th^ percentiles of the distribution.

17. **P. 7, l. 136: I’m not sure what is meant by guaranteed here. I take it this means all were observed in the simulation, but there is not actually a probabilistic guarantee that would hold in general?**

We agree with the reviewer that the term “guaranteed” is not suitable. We have therefore simplified the paragraph by removing this sentence, which was not essential.

*“In the first scenario, we assumed a rich sampling design in a population of 120 individuals (i.e. 60 infected individuals), with daily observations from infection to clearance for each individual.” Lines 135-135*

18. **P. 7, l. 146: This should be re-numbered as Figure 2.**

We thank the reviewer for this comment; the figure numbering has been adjusted accordingly.

19. **P. 8, Eqn 2: n+ and n- should be defined here rather than below equation 4. They should also be indexed by i as they may vary from person to person.**

We thank the reviewer for their careful reading; this has been corrected.

20. **P. 8, ll. 165–166: Can you clarify whether P_inf_ represents a population parameter or an estimate here?**

We have clarified the sentence: "$P_{inf}$ *denotes a population parameter corresponding to the estimated proportion of infected individuals*” *Lines 165-166*

21. **P. 8, l. 169: Can you clarify the specific conditional probability represented by P_TN_?**

We have clarified the meaning of P_TN._

*“with* $P_{TN}$ *denotes the probability of true negative, the probability of testing negative given that the individual is not infected, and was fixed to 0.9998 in line with the high specificity of PCR testing.” Lines 168-169*

22. **P. 11, Tbl. 1: It would help to add percentages of each group represented by the number in each category (e.g., female % for each column, % of individuals with 1 test, etc.)**

Thank you for pointing this out; we have added the percentages to make it easier to compare the subgroups.

23. **P. 11, Tbl. 1: For the bottom section, it’s not clear to me how these can be per test values, but add up to the total number of individuals in the group. Perhaps I am missing something, but could this be clarified?**

We agree that this was misleading and redundant with respect to Figure 2. We have therefore removed this row from Table 1.

24. **P. 12: It would be helpful to have figures showing the average widths of the intervals, so that their coverage performance can be assessed in light of the uncertainty they represent.**

We agree with this comment, so we have included the 95% confidence (and credibility) intervals for all inferences from the simulation study in the Supplementary Materials (see comment number 10).

25. **P. 14, l. 279: I think it is an overstatement to call this “minimal bias”; and the issues with inference should be noted here as well.**

We believe that the estimation bias is indeed present, but it is not problematic for identifying differences between subgroups. First, it is relatively limited (less than +3.6%, i.e., approximately +0.5 day; Figure 3 and S5). In addition, it shows very little variability across simulated datasets and is consistently in the same direction (Figure S6C); the interquartile range of the boxplot is narrow (between +1.7% and +5.1%). We have replaced “minimal bias” with “limited bias”.

26. **P. 14: I think Table S3 would be useful in the main text rather than the supplement as it is the key result of the data analysis.**

We thank the reviewer for this comment. We moved the table of parameter estimates to the main text and is now presented as Table 2.

27. **Figure 4: The caption would be clearer if it read “percentage of PCR tests that were negative” rather than “percentage of negative tests”**

The figure caption has been modified in line with your comment.

28. **Figure 6: can you clarify how the uncertainty on the trajectories is computed in this figure? What assumptions, if any, are made about the independence of the parameter estimates?**

For each parameter and subgroup, we draw 10,000 replicates from a multivariate Gaussian distribution (using the *rmvnorm* function from the R package *mvtnorm*, version 1.3-3), with mean vector μ (the population parameter estimates) and covariance given by the Fisher information matrix computed by Monolix. For each of the 10,000 replicates within each subgroup, we reconstruct the viral kinetics. The mean of these 10,000 trajectories, along with the 5th and 95th percentiles, are used to define the mean trajectory and the lower and upper bounds of the 95% confidence interval. In this way, we account for both parameter estimation uncertainty and the correlation structure between parameter estimates.

The full description and associated scripts are available on GitHub in the external validation scripts (1).

**Figure S4: the scale is unclear: is it both days and Ct values?**

We have revised the y-axis label of the figure to indicate that the unit varies depending on the parameter, and this is also specified in the legend and in the parameter labels (x-axis).

**References**

1.	Beaulieu M. Code and resources for simulation [R] [Internet]. 2025. Available from: https://github.com/Germante1/Code_SARS_CoV_viral_dynamics_in_community

2.	Jittamala P, Schilling WHK, Watson JA, Luvira V, Siripoon T, Ngamprasertchai T, et al. Clinical Antiviral Efficacy of Remdesivir in Coronavirus Disease 2019: An Open-Label, Randomized Controlled Adaptive Platform Trial (PLATCOV). The Journal of Infectious Diseases. 2023 Nov;228(10):1318–25. doi:10.1093/infdis/jiad275

3.	Gavina K, Franco LC, Robinson CM, Hymas W, Lei GS, Sinclair W, et al. Standardization of SARS-CoV-2 Cycle Threshold Values: Multisite Investigation Evaluating Viral Quantitation across Multiple Commercial COVID-19 Detection Platforms. Microbiology Spectrum. 2023 Jan 18;11(1):e04470-22. doi:10.1128/spectrum.04470-22

4.	Eyre DW, Taylor D, Purver M, Chapman D, Fowler T, Pouwels KB, et al. Effect of Covid-19 Vaccination on Transmission of Alpha and Delta Variants. New England Journal of Medicine. 2022 Feb 23;386(8):744–56. doi:10.1056/NEJMoa2116597

5.	Elie B, Roquebert B, Sofonea MT, Trombert-Paolantoni S, Foulongne V, Guedj J, et al. Variant-specific SARS-CoV-2 within-host kinetics. Journal of Medical Virology. 2022;94(8):3625–33. doi:10.1002/jmv.27757

6.	Cosentino G, Bernard M, Ambroise J, Giannoli JM, Guedj J, Débarre F, et al. SARS-CoV-2 viral dynamics in infections with Alpha and Beta variants of concern in the French community. Journal of Infection. 2022 Jan 1;84(1):94–118. doi:10.1016/j.jinf.2021.07.031 PubMed PMID: 34329672.

7.	Yang Y, Guo L, Yuan J, Xu Z, Gu Y, Zhang J, et al. Viral and antibody dynamics of acute infection with SARS-CoV-2 omicron variant (B.1.1.529): a prospective cohort study from Shenzhen, China. The Lancet Microbe. 2023 Aug 1;4(8):e632–41. doi:10.1016/S2666-5247(23)00139-8 PubMed PMID: 37459867.

8.	Wongnak P, Schilling WHK, Jittamala P, Boyd S, Luvira V, Siripoon T, et al. Temporal changes in SARS-CoV-2 clearance kinetics and the optimal design of antiviral pharmacodynamic studies: an individual patient data meta-analysis of a randomised, controlled, adaptive platform study (PLATCOV). Lancet Infect Dis. 2024 Apr 24;S1473-3099(24)00183-X. doi:10.1016/S1473-3099(24)00183-X PubMed PMID: 38677300.

9.	ameli.fr. Chiffres clés en France — Data vaccin Covid [Internet]. 2022. Available from: https://datavaccin-covid.ameli.fr/pages/synthese/

10.	Galmiche S, Cortier T, Charmet T, Schaeffer L, Chény O, Von Platen C, et al. SARS-CoV-2 incubation period across variants of concern, individual factors, and circumstances of infection in France: a case series analysis from the ComCor study. The Lancet Microbe. 2023 Jun;4(6):e409–17. doi:10.1016/S2666-5247(23)00005-8

11.	Hay JA, Kissler SM, Fauver JR, Mack C, Tai CG, Samant RM, et al. Quantifying the impact of immune history and variant on SARS-CoV-2 viral kinetics and infection rebound: A retrospective cohort study. eLife. 2022 Nov 16;11:e81849. doi:10.7554/eLife.81849

12.	Kissler SM, Fauver JR, Mack C, Olesen SW, Tai C, Shiue KY, et al. Viral dynamics of acute SARS-CoV-2 infection and applications to diagnostic and public health strategies. PLOS Biology. 2021 Jul 12;19(7):e3001333. doi:10.1371/journal.pbio.3001333

13.	Killingley B, Mann AJ, Kalinova M, Boyers A, Goonawardane N, Zhou J, et al. Safety, tolerability and viral kinetics during SARS-CoV-2 human challenge in young adults. Nat Med. 2022 May;28(5):1031–41. doi:10.1038/s41591-022-01780-9
