# Supplementary material for: Quantitative analysis of massive SARS-CoV-2 testing in the community in France in 2021–2022 reveals the associations of variant, vaccination, and age with viral dynamics in symptomatic individuals
Source: PLoS Comput Biol. 2026 Jul 27;22(7):e1013811. doi: 10.1371/journal.pcbi.1013811 (PMC13426954; doi:10.1371/journal.pcbi.1013811)
Supplement: Attachment — Submitted filename: Response_to_Reviewer_auresp_2.docx [file pcbi.1013811.s023.docx]

**Review:** Quantitative analysis of massive SARS-CoV-2 testing in the community in France in 2021-2022 reveals the associations of variant, vaccination, and age with viral dynamics in symptomatic individuals

**Responses to referees and editor**

We would like to thank the reviewers and the Editor for their comments, which helped us further improve the manuscript. We have addressed all remaining points raised during this round of review and have carefully reviewed the data retrieval process to ensure the availability of the data and associated resources. Please find below our point-by-point response.

**Reviewer #1: James A Watson**

No remarks.

**Reviewer #2:**

Thank you to the authors for their attention to the prior reviews and their careful revision of the manuscript. Given the clarified focus of the manuscript and the careful languages, most of my concerns from the prior review have been addressed. I have only minor comments remaining:

**Minor points:**

1. **P. 10, l. 207: Replace “effects” with associations.**

The sentence has been modified in line with your comment.

2. **Pp. 15–16: Given the consistently poor performance of the frequently confidence intervals in the simulation, I think it is misleading to provide them in the data analysis.**

We thank you for your suggestion. We however think it is better to show the 95% confidence intervals. As shown in Figure S6C, in Scenario 3 (which mimics the real-world data situation), the coverage rate for the peak viral load is satisfactory and, although the coverage rate is not 95% for the clearance period, most of the confidence intervals for this parameter do not include the simulation value within 0.2 day. To address your comment, we have added a statement in the Discussion highlighting the costs of the frequentist approach.

Modifications made in the Discussion section:

*“We here decided to use a pragmatic strategy, focusing on modelling confirmed infections using a frequentist inference framework. The model simulations show that this choice comes at the cost of a reduced accuracy and precision for parameter estimates, in particular for the pre-symptomatic period, however the magnitude of these effects was limited for the parameters driving the post-symptomatic period, i.e., peak viral load and time to viral clearance (****Figure S5, S6C****). “* *Lines 344-348*

3. **Pp. 15–16: It would be interesting to see if confidence intervals for the differences between covariate levels showed better coverage than CIs for the parameters themselves. I do not know how feasible this is with the model as currently constructed, so it is not necessary at this point.**

We thank you for this very remark. Unfortunately, and as noted by the reviewer, this is not feasible in the model as the simulation were not designed to estimate differences. Consistent with this approach, we do not test for these differences in the real data analysis.
