# Supplementary material for: Quantitative analysis of massive SARS-CoV-2 testing in the community in France in 2021–2022 reveals the associations of variant, vaccination, and age with viral dynamics in symptomatic individuals
Source: PLoS Comput Biol. 2026 Jul 27;22(7):e1013811. doi: 10.1371/journal.pcbi.1013811 (PMC13426954; doi:10.1371/journal.pcbi.1013811)
Supplement: Peer review history

# Peer review: Decision Letter 0

**DOI:** 10.1371/journal.pcbi.1013811.r001

**Authors:** Rustom Antia, Stacey D. Finley

11 Feb 2026

PCOMPBIOL-D-25-02547

The analysis of a large dataset of PCR-test results in the community reveals the impact of age, variants and vaccination status on SARS-CoV-2 viral dynamics

PLOS Computational Biology

Dear Dr. Beaulieu,

Thank you for submitting your manuscript to PLOS Computational Biology. After careful consideration, we feel that it has merit but does not fully meet PLOS Computational Biology's publication criteria as it currently stands. Therefore, we invite you to submit a revised version of the manuscript that addresses the points raised during the review process.

Please submit your revised manuscript by Apr 13 2026 11:59PM. If you will need more time than this to complete your revisions, please reply to this message or contact the journal office at ploscompbiol@plos.org. When you're ready to submit your revision, log on to https://www.editorialmanager.com/pcompbiol/ and select the 'Submissions Needing Revision' folder to locate your manuscript file.

Please include the following items when submitting your revised manuscript:

* A letter that responds to each point raised by the editor and reviewer(s). You should upload this letter as a separate file labeled 'Response to Reviewers'. This file does not need to include responses to formatting updates and technical items listed in the 'Journal Requirements' section below.

* A marked-up copy of your manuscript that highlights changes made to the original version. You should upload this as a separate file labeled 'Revised Manuscript with Track Changes'.

* An unmarked version of your revised paper without tracked changes. You should upload this as a separate file labeled 'Manuscript'.

If you would like to make changes to your financial disclosure, competing interests statement, or data availability statement, please make these updates within the submission form at the time of resubmission. Guidelines for resubmitting your figure files are available below the reviewer comments at the end of this letter

We look forward to receiving your revised manuscript.

Kind regards,

Rustom Antia

Academic Editor

PLOS Computational Biology

Stacey Finley

Section Editor

PLOS Computational Biology

**Additional Editor Comments:**

First, apologies for the time taken to get the reviews. Both reviewers found the paper addresses an important (ambitious) set of questions but indicated that the paper requires substantial revisions. If you feel more time is required for revision please let us know.

**Journal Requirements:**

If the reviewer comments include a recommendation to cite specific previously published works, please review and evaluate these publications to determine whether they are relevant and should be cited. There is no requirement to cite these works unless the editor has indicated otherwise.

1) We ask that a manuscript source file is provided at Revision. Please upload your manuscript file as a .doc, .docx, .rtf or .tex. If you are providing a .tex file, please upload it under the item type u2018LaTeX Source Fileu2019 and leave your .pdf version as the item type u2018Manuscriptu2019.

2) Please upload all main figures as separate Figure files in .tif or .eps format. For more information about how to convert and format your figure files please see our guidelines:

https://journals.plos.org/ploscompbiol/s/figures

3) We have noticed that you have uploaded Supporting Information files, but you have not included a list of legends. Please add a full list of legends for your Supporting Information files after the references list.

4) In the online submission form, you indicated that Human data and associated metadata are not publicly available. Requests for access for research purposes may be addressed to Biogroup at gina.cosentino@biogroup.fr. All PLOS journals now require all data underlying the findings described in their manuscript to be freely available to other researchers, either

1. In a public repository

2. Within the manuscript itself

3. Uploaded as supplementary information.

This policy applies to all data except where public deposition would breach compliance with the protocol approved by your research ethics board. If your data cannot be made publicly available for ethical or legal reasons (e.g., public availability would compromise patient privacy), please explain your reasons by return email and your exemption request will be escalated to the editor for approval. Your exemption request will be handled independently and will not hold up the peer review process, but will need to be resolved should your manuscript be accepted for publication. One of the Editorial team will then be in touch if there are any issues.

5) Please amend your detailed Financial Disclosure statement. This is published with the article. It must therefore be completed in full sentences and contain the exact wording you wish to be published.

1) State the initials, alongside each funding source, of each author to receive each grant. For example: "This work was supported by the National Institutes of Health (####### to AM; ###### to CJ) and the National Science Foundation (###### to AM)."

2) State what role the funders took in the study. If the funders had no role in your study, please state: "The funders had no role in study design, data collection and analysis, decision to publish, or preparation of the manuscript."

3) If any authors received a salary from any of your funders, please state which authors and which funders..

If you did not receive any funding for this study, please simply state: u201cThe authors received no specific funding for this work.u201d

**Reviewers' comments:**

Reviewer's Responses to Questions

**Comments to the Authors:**

**Please note here if the review is uploaded as an attachment.**

Reviewer #1: This work uses routinely collected PCR testing data to infer the viral kinetics of SARS-CoV-2 and the effects of variant, age and sex. The authors have access to a very large dataset from a private laboratory with nearly 400K records with basic clinical data. They pair the observed CT values with the self-reported time from symptom onset to recover the population viral kinetics. This is an important demonstration of the value of these routinely collected data. I enjoyed reading the paper.

My main comments are:

• I think the paper would benefit from some more discussion/thinking around potential biases in the data collection. What do the authors think might be a reasonable causal structure for the data? For example, do they think it is possible that disease severity is a confounder? Suppose that older patients are generally followed-up more closely when they are sicker and therefore the observed slower clearance in older patients is in fact driven by severity rather than age? It would be useful to say what your causal assumptions are, maybe with the use of a DAG.

• Validation on an external dataset. For this research to be fully convincing, I would ideally hope to see the inferred parameters validated using an external dataset where patients have been prospectively followed. Ideally this would a dataset such as the US NBA (https://journals.plos.org/plosbiology/article?id=10.1371/journal.pbio.3001333), but if there is no good equivalent for this timeframe then just a prospective study in symptomatic patients (eg PLATCOV studies)? If the authors could show that their inferred parameters approximate well parameters inferred from more detailed data, then this would give a much stronger result. This is especially important as the authors don’t use the best method (fully Bayesian) because of the computational issues. It’s clear from the simulations that the Monolix method gives biased inference.

• Are the parameters inferred believable? This links to the point above and may reflect my misunderstanding. In table S3, the peak viral load estimates are between 14 and 17 on the CT scale. This seems very low (ie very high viral load)? Given that this is the population average by subgroup, I find the values hard to believe. The prior is 25 which seems more reasonable. Very few CT values in Fig S8 are below 14. Does this represent an issue in the inference?

• No code or data. Will the authors make the code available? And can they make the CT data available as well? Given that this is a computational journal, code/data availability is very important for reproducibility.

Minor points:

•Have the authors thought about using this approach for other viral infections? Notably ‘flu

•Equation 2 notation is confusing as f is used for the model structure and the normal pdf. Could just use another letter for the model, e.g. g(x)?

•Is there any temporal trend in clearance values not explained by variant? Eg as suggested by the PLATCOV study (https://pubmed.ncbi.nlm.nih.gov/38677300/). This could be looked at by comparing model random effect estimates with calendar date.

Reviewer #2: See attached review

**********

**Have the authors made all data and (if applicable) computational code underlying the findings in their manuscript fully available?**

The PLOS Data policy requires authors to make all data and code underlying the findings described in their manuscript fully available without restriction, with rare exception (please refer to the Data Availability Statement in the manuscript PDF file). The data and code should be provided as part of the manuscript or its supporting information, or deposited to a public repository. For example, in addition to summary statistics, the data points behind means, medians and variance measures should be available. If there are restrictions on publicly sharing data or code —e.g. participant privacy or use of data from a third party—those must be specified.

Reviewer #1: **No:**  no details provided

Reviewer #2: Yes

**********

PLOS authors have the option to publish the peer review history of their article (what does this mean?). If published, this will include your full peer review and any attached files.

If you choose “no”, your identity will remain anonymous but your review may still be made public.

**Do you want your identity to be public for this peer review?**  For information about this choice, including consent withdrawal, please see our Privacy Policy.

Reviewer #1: **Yes:**  James A Watson

Reviewer #2: No

[NOTE: If reviewer comments were submitted as an attachment file, they will be attached to this email and accessible via the submission site. Please log into your account, locate the manuscript record, and check for the action link "View Attachments". If this link does not appear, there are no attachment files.]

**Figure resubmission:**

While revising your submission, we strongly recommend that you use PLOS’s NAAS tool (https://ngplosjournals.pagemajik.ai/artanalysis) to test your figure files. NAAS can convert your figure files to the TIFF file type and meet basic requirements (such as print size, resolution), or provide you with a report on issues that do not meet our requirements and that NAAS cannot fix.

After uploading your figures to PLOS’s NAAS tool - https://ngplosjournals.pagemajik.ai/artanalysis, NAAS will process the files provided and display the results in the "Uploaded Files" section of the page as the processing is complete. If the uploaded figures meet our requirements (or NAAS is able to fix the files to meet our requirements), the figure will be marked as "fixed" above. If NAAS is unable to fix the files, a red "failed" label will appear above. When NAAS has confirmed that the figure files meet our requirements, please download the file via the download option, and include these NAAS processed figure files when submitting your revised manuscript.

**Reproducibility:**

To enhance the reproducibility of your results, we recommend that authors of applicable studies deposit laboratory protocols in protocols.io, where a protocol can be assigned its own identifier (DOI) such that it can be cited independently in the future. Additionally, PLOS ONE offers an option to publish peer-reviewed clinical study protocols. Read more information on sharing protocols at https://plos.org/protocols?utm_medium=editorial-email&utm_source=authorletters&utm_campaign=protocols

Attachment Submitted filename: Review.docx

# Author comment: Author response to Decision Letter 1

**DOI:** 10.1371/journal.pcbi.1013811.r002

18 May 2026

Attachment Submitted filename: Response_to_Reviewer.docx

# Peer review: Decision Letter 1

**DOI:** 10.1371/journal.pcbi.1013811.r003

**Authors:** Rustom Antia, Stacey D. Finley

1 Jul 2026

PCOMPBIOL-D-25-02547R1

Quantitative analysis of massive SARS-CoV-2 testing in the community in France in 2021-2022 reveals the associations of variant, vaccination, and age with viral dynamics in symptomatic individuals

PLOS Computational Biology

Dear Dr. Beaulieu,

Thank you for submitting your manuscript to PLOS Computational Biology. After careful consideration, we feel that it has merit but does not fully meet PLOS Computational Biology's publication criteria as it currently stands. Therefore, we invite you to submit a revised version of the manuscript that addresses the points raised during the review process.

Please submit your revised manuscript by Aug 31 2026 11:59PM. If you will need more time than this to complete your revisions, please reply to this message or contact the journal office at ploscompbiol@plos.org. When you're ready to submit your revision, log on to https://www.editorialmanager.com/pcompbiol/ and select the 'Submissions Needing Revision' folder to locate your manuscript file.

Please include the following items when submitting your revised manuscript:

* A letter that responds to each point raised by the editor and reviewer(s). You should upload this letter as a separate file labeled 'Response to Reviewers'. This file does not need to include responses to formatting updates and technical items listed in the 'Journal Requirements' section below.

* A marked-up copy of your manuscript that highlights changes made to the original version. You should upload this as a separate file labeled 'Revised Manuscript with Track Changes'.

* An unmarked version of your revised paper without tracked changes. You should upload this as a separate file labeled 'Manuscript'.

If you would like to make changes to your financial disclosure, competing interests statement, or data availability statement, please make these updates within the submission form at the time of resubmission. Guidelines for resubmitting your figure files are available below the reviewer comments at the end of this letter.

As the corresponding author, your ORCID iD is verified in the submission system and will appear in the published article. PLOS supports the use of ORCID, and we encourage all coauthors to register for an ORCID iD and use it as well. Please encourage your coauthors to verify their ORCID iD within the submission system before final acceptance, as unverified ORCID iDs will not appear in the published article. Only the individual author can complete the verification step; PLOS staff cannot verify ORCID iDs on behalf of authors.

We look forward to receiving your revised manuscript.

Kind regards,

Rustom Antia

Academic Editor

PLOS Computational Biology

Stacey Finley

Section Editor

PLOS Computational Biology

**Additional Editor Comments (if provided):**

Sorry for the time it has taken to get comments from the reviewers. The paper looks very good. Please address the minor comments in the review.

**Journal Requirements:**

If the reviewer comments include a recommendation to cite specific previously published works, please review and evaluate these publications to determine whether they are relevant and should be cited. There is no requirement to cite these works unless the editor has indicated otherwise.

1) We have noticed that you have uploaded Supporting Information files, but you have not included a list of legends. Please add a full list of legends for your Supporting Information files after the references list.

2)Thank you for stating "The data underlying this study consist of individual-level human data collected in the context of routine clinical care and are not publicly available due to ethical, legal, and regulatory restrictions, including compliance with data protection regulations (e.g., GDPR). The data are owned and controlled by Biogroup, which serves as the independent data controller. While some employees of the company are co-authors of this study, data access requests are handled independently through the company’s established data access procedures. Data are available upon reasonable request to qualified researchers for legitimate scientific purposes, subject to review and approval in accordance with applicable regulations and the company's data access policies.Requests for access should be directed to: Biogroup Email: recherche.nouveauprojet@biogroup.fr." However, we were unable to access the data using the information provided. Please review and update the Data Availability Statement to ensure that the underlying data are publicly accessible and that all accession numbers, links, or repository details are correct and functional.

**Reviewers' comments:**

Reviewer's Responses to Questions

**Comments to the Authors:**

**Please note here if the review is uploaded as an attachment.**

Reviewer #2: Thank you to the authors for their attention to the prior reviews and their careful revision of the manuscript. Given the clarified focus of the manuscript and the careful languages, most of my concerns from the prior review have been addressed. I have only minor comments remaining:

P. 10, l. 207: Replace “effects” with associations

Pp. 15–16: Given the consistently poor performance of the frequently confidence intervals in the simulation, I think it is misleading to provide them in the data analysis.

Pp. 15–16: It would be interesting to see if confidence intervals for the differences between covariate levels showed better coverage than CIs for the parameters themselves. I do not know how feasible this is with the model as currently constructed, so it is not necessary at this point.

**********

**Have the authors made all data and (if applicable) computational code underlying the findings in their manuscript fully available?**

The PLOS Data policy requires authors to make all data and code underlying the findings described in their manuscript fully available without restriction, with rare exception (please refer to the Data Availability Statement in the manuscript PDF file). The data and code should be provided as part of the manuscript or its supporting information, or deposited to a public repository. For example, in addition to summary statistics, the data points behind means, medians and variance measures should be available. If there are restrictions on publicly sharing data or code —e.g. participant privacy or use of data from a third party—those must be specified.

Reviewer #2: **No:**  While the code is available, the authors have stated that they are restricted from making the underlying data publicly available.

**********

PLOS authors have the option to publish the peer review history of their article (what does this mean?). If published, this will include your full peer review and any attached files.

If you choose “no”, your identity will remain anonymous but your review may still be made public.

**Do you want your identity to be public for this peer review?**  For information about this choice, including consent withdrawal, please see our Privacy Policy.

Reviewer #2: No

[NOTE: If reviewer comments were submitted as an attachment file, they will be attached to this email and accessible via the submission site. Please log into your account, locate the manuscript record, and check for the action link "View Attachments". If this link does not appear, there are no attachment files.]

**Figure resubmission:**

-->While revising your submission, we strongly recommend that you use PLOS’s NAAS tool (https://ngplosjournals.pagemajik.ai/artanalysis) to test your figure files. NAAS can convert your figure files to the TIFF file type and meet basic requirements (such as print size, resolution), or provide you with a report on issues that do not meet our requirements and that NAAS cannot fix.

After uploading your figures to PLOS’s NAAS tool - https://ngplosjournals.pagemajik.ai/artanalysis, NAAS will process the files provided and display the results in the "Uploaded Files" section of the page as the processing is complete. If the uploaded figures meet our requirements (or NAAS is able to fix the files to meet our requirements), the figure will be marked as "fixed" above. If NAAS is unable to fix the files, a red "failed" label will appear above. When NAAS has confirmed that the figure files meet our requirements, please download the file via the download option, and include these NAAS processed figure files when submitting your revised manuscript.

**Reproducibility:**

To enhance the reproducibility of your results, we recommend that authors of applicable studies deposit laboratory protocols in protocols.io, where a protocol can be assigned its own identifier (DOI) such that it can be cited independently in the future. Additionally, PLOS ONE offers an option to publish peer-reviewed clinical study protocols. Read more information on sharing protocols at https://plos.org/protocols?utm_medium=editorial-email&utm_source=authorletters&utm_campaign=protocols

# Author comment: Author response to Decision Letter 2

**DOI:** 10.1371/journal.pcbi.1013811.r004

6 Jul 2026

Attachment Submitted filename: Response_to_Reviewer_auresp_2.docx

# Editor report: Decision Letter 2

**DOI:** 10.1371/journal.pcbi.1013811.r005

**Authors:** Rustom Antia, Stacey D. Finley

8 Jul 2026

Dear Mr Beaulieu,

We are pleased to inform you that your manuscript 'Quantitative analysis of massive SARS-CoV-2 testing in the community in France in 2021-2022 reveals the associations of variant, vaccination, and age with viral dynamics in symptomatic individuals' has been provisionally accepted for publication in PLOS Computational Biology.

Before your manuscript can be formally accepted you will need to complete some formatting changes, which you will receive in a follow up email. A member of our team will be in touch with a set of requests.

Please note that your manuscript will not be scheduled for publication until you have made the required changes, so a swift response is appreciated.

IMPORTANT: The editorial review process is now complete. PLOS will only permit corrections to spelling, formatting or significant scientific errors from this point onwards. Requests for major changes, or any which affect the scientific understanding of your work, will cause delays to the publication date of your manuscript.

Should you, your institution's press office or the journal office choose to press release your paper, you will automatically be opted out of early publication. We ask that you notify us now if you or your institution is planning to press release the article. All press must be co-ordinated with PLOS.

Thank you again for supporting Open Access publishing; we are looking forward to publishing your work in PLOS Computational Biology.

Best regards,

Rustom Antia

Academic Editor

PLOS Computational Biology

Stacey Finley

Section Editor

PLOS Computational Biology

***********************************************************

# Editor report: Acceptance letter

**DOI:** 10.1371/journal.pcbi.1013811.r006

**Authors:** Rustom Antia, Stacey D. Finley

PCOMPBIOL-D-25-02547R2

Quantitative analysis of massive SARS-CoV-2 testing in the community in France in 2021-2022 reveals the associations of variant, vaccination, and age with viral dynamics in symptomatic individuals

Dear Dr Beaulieu,

I am pleased to inform you that your manuscript has been formally accepted for publication in PLOS Computational Biology. Your manuscript is now with our production department and you will be notified of the publication date in due course.

The corresponding author will soon be receiving a typeset proof for review, to ensure errors have not been introduced during production. Please review the PDF proof of your manuscript carefully, as this is the last chance to correct any errors. Please note that major changes, or those which affect the scientific understanding of the work, will likely cause delays to the publication date of your manuscript.

Soon after your final files are uploaded, unless you have opted out, the early version of your manuscript will be published online. The date of the early version will be your article's publication date. The final article will be published to the same URL, and all versions of the paper will be accessible to readers.

For Research, Software, and Methods articles, you will receive an invoice from PLOS for your publication fee after your manuscript has reached the completed accept phase. If you receive an email requesting payment before acceptance or for any other service, this may be a phishing scheme. Learn how to identify phishing emails and protect your accounts at https://explore.plos.org/phishing.

Thank you again for supporting PLOS Computational Biology and open-access publishing. We are looking forward to publishing your work!

With kind regards,

Janani Seenivasan

PLOS Computational Biology | Carlyle House, Carlyle Road, Cambridge CB4 3DN | United Kingdom ploscompbiol@plos.org | Phone +44 (0) 1223-442824 | ploscompbiol.org | @PLOSCompBiol
